# GABAergic Control of Nigrostriatal and Mesolimbic Dopamine in the Rat Brain

**DOI:** 10.3389/fnbeh.2018.00038

**Published:** 2018-03-14

**Authors:** Susanne Nikolaus, Hans-Jörg Wittsack, Markus Beu, Christina Antke, Maria A. De Souza Silva, Frijthof Wickrath, Anja Müller-Lutz, Joseph P. Huston, Gerald Antoch, Hans-Wilhelm Müller, Hubertus Hautzel

**Affiliations:** ^1^Clinic of Nuclear Medicine, University Hospital Düsseldorf, Düsseldorf, Germany; ^2^Department of Diagnostic and Interventional Radiology, Medical Faculty, University Düsseldorf, Düsseldorf, Germany; ^3^Center for Behavioural Neuroscience, Institute of Experimental Psychology, Heinrich-Heine University, Düsseldorf, Germany

**Keywords:** D_2/3_ receptor, [^123^I]IBZM, GABA_A_ receptor, muscimol, bicuculline

## Abstract

**Purpose:** The present study assessed the effects of the GABA_A_ receptor (R) agonist muscimol (MUS), and the GABA_A_R antagonist bicuculline (BIC) on neocortical and subcortical radioligand binding to dopamine D_2/3_Rs in relation to motor and exploratory behaviors in the rat.

**Methods:** D_2/3_R binding was measured with small animal SPECT in baseline and after challenge with either 1 mg/kg MUS or 1 mg/kg BIC, using [^123^I]IBZM as radioligand. Motor/exploratory behaviors were assessed for 30 min in an open field prior to radioligand administration. Anatomical information was gained with a dedicated small animal MRI tomograph. Based on the Paxinos rat brain atlas, regions of interest were defined on SPECT-MRI overlays. Estimations of the binding potentials in baseline and after challenges were obtained by computing ratios of the specifically bound compartments to the cerebellar reference region.

**Results:** After MUS, D_2/3_R binding was significantly reduced in caudateputamen, nucleus accumbens, thalamus, substania nigra/ventral tegmental area, and posterior hippocampus relative to baseline (0.005 ≤ *p* ≤ 0.012). In all these areas, except for the thalamus, D_2/3_R binding was negatively correlated with grooming in the first half and positively correlated with various motor/exploratory behaviors in the second half of the testing session. After BIC, D_2/3_R binding was significantly elevated in caudateputamen (*p* = 0.022) and thalamus (*p* = 0.047) relative to baseline. D_2/3_R binding in caudateputamen and thalamus was correlated negatively with sitting duration and sitting frequency and positively with motor/exploratory behaviors in the first half of the testing time.

**Conclusions:** Findings indicate direct GABAergic control over nigrostriatal and mesolimbic dopamine levels in relation to behavioral action. This may be of relevance for neuropsychiatric conditions such as anxiety disorder and schizophrenia, which are characterized by both dopaminergic and GABAergic dysfunction.

## Introduction

In the nigrostriatal system, the neostriatum or caudateputamen (CP) receives glutamatergic (GLUergic) afferents from neocortex [motor cortex (MC), somatosensory cortex, frontal cortex (FC), prefronal cortex (PFC)] and thalamus (THAL; Johnson et al., 1968; Jones et al., 1977; Künzle, 1977; Jayaraman, 1985) as well as dopaminergic (DAergic) afferents from the pars compacta of the substantia nigra (SNc; Gerfen et al., 1987). In turn, it sends DAergic efferents to THAL, neocortex, and back to the SN (Gerfen, 1984; Tomasi and Volkow, 2013). In the mesolimbic system, the ventral striatum or nucleus accumbens (NAC), receives GLUergic imput from cortical regions, hippocampus (HIPP), amygdala, and cingulate (CING) as well as DAergic input from ventral tegmental area (VTA) and the parafascilular nucleus of the THAL (Powell and Leman, 1976; Baleydier and Mauguiere, 1980; Hara et al., 1989; Sesack et al., 1989; Ishikawa and Nakamura, 2006), with DAergic neurons projecting back to limbic regions including the HIPP (Nazari-Serenjeh et al., 2011).

The DAergic system is under inhibitory γ-amino butyric acidergic (GABAergic) control (Precht and Yoshida, 1971). From the CP, GABAergic projections run either directly or via the external part of the globus pallidus (GPe) and the subthalamic nucleus (STN) to the internal part of the globus pallidus (GPi) and to the pars reticulata of the SN (SNr). From GPi and SNr, further GABAergic efferents project to THAL (ventral anterior, ventrolateral, dorsomedial, and centromedian nucleus), pedunculopontine nucleus, inferior and superior colliculus, and periaqueducatal gray (Kuo and Carpenter, 1973; Graybiel and Ragsdale, 1979; Herkenham, 1979; Coimbra and Brandao, 1993). In addition, the CP is inhibited by GABAergic mircrocircuits, which are formed by fast-spiking interneurons and collaterals of descending projection neurons (Groves, 1983). The NAC receives GABAergic input from the PFC (Lee et al., 2014), and, in turn, sends GABAergic efferents back to the VTA as well as to the ventral GP and from the GP to the dorsomedial thalamic nucleus (Ueki et al., 1977; Yamamoto et al., 1983). DA neurons serve to modulate the GABAergic system: in the direct pathway, DA exerts an inhibitory effect on those GABAergic neurons, which project to the GPe, leading to a net disinhibition of the THAL, whereas in the indirect pathway, DA stimulates GABAergic projections to GPi and SNr with a net inhibitory effect on the THAL. The THAL, in turn, sends GLUergic efferents to MC, FC, PFC, and CING.

The dysfunction of the GABA_A_R subtype is associated with numerous psychiatric conditions including schizophrenia, anxiety disorders and autism spectrum disorders (Cellot and Cherubini, 2014; Nikolaus et al., 2014b). Intracerebral effects of the GABA_A_R agonist muscimol (MUS; 5-aminomethyl-isoxazol-3-ol) and the GABA_A_R antagonist bicuculline (BIC; (6R)-6-[(5S)-6-methyl-5,6,7,8-tetrahydro[1,3]dioxolo[4,5-g]isoquinolin-5-yl]furo[3,4-e][1,3]benzodioxol-8(6H)-one) on extracellular DA are region-specific: MUS infusions into the PFC (0.1 and 1 mM, Matsumoto et al., 2003) and into the VTA (10–40 μM, Westerink et al., 1996, 1998) reduced DA concentrations in CP (Westerink et al., 1996; Matsumoto et al., 2003) and PFC (Westerink et al., 1998). In contrast, MUS infusion into SN (10 μM, Santiago and Westerink, 1992) and GP (100 μM, Cobb and Abercrombie, 2002, 2003) increased DA levels in CP and SN, respectively. Furthermore, application into NAC (250 μM, Yoshida et al., 1997; 0.5 nM, Aono et al., 2008) and VTA (10 and 100 μM, Klitenick et al., 1992) elevated DA in these regions. However, no effect was observed on prefrontal DA after prefrontal infusion of 50 and 500 μM MUS (Santiago et al., 1993).

BIC infusions into SN (50 μM, Santiago and Westerink, 1992; Westerink et al., 1992), VTA (200 μM, Ikemoto et al., 1997), and PFC (50 and 100 μM, Karreman and Moghaddam, 1996; 30 and 100 μM, Matsumoto et al., 2003) elevated DA concentrations in SN and CP (Santiago and Westerink, 1992; Westerink et al., 1992) as well as NAC (Ikemoto et al., 1997) and PFC (Karreman and Moghaddam, 1996; Matsumoto et al., 2003). Similarly, applications into CP (100 μM, Smolders et al., 1995) and NAC (25, 50, or 100 μM, Yan, 1999; 50 pmol, Aono et al., 2008) increased DA levels in these regions.

We have previously shown that MUS but not BIC reduced radioligand binding to the neostriatal D_2_R-like subtype relative to baseline (BAS) and to vehicle. Moreover, motor/exploratory behaviors were diminished after MUS, but elevated after BIC relative to vehicle (Nikolaus et al., 2017). These effects of MUS and BIC prompted us to assess binding to the D_2_R-like subtype separately in CP and NAC as well as in other cortical and subcortical regions including THAL, SN/VTA, FC, MC, parietal cortex (PC), anterodorsal hippocampus (aHIPP), and posterior hippocampus (pHIPP). In the previous study, for each rat, small volumes of interest (VOIs) had been defined around the neostriatal hot spots of maximum radioactivity accumulations (Nikolaus et al., 2017). This mode of analysis has proven sufficient for a comparatively large region with a high density of D_2_R-like binding sites such as the striatum. However, it may be not valid to determine radioligand binding in smaller regions such as SN/VTA, or in cortical or limbic regions with lower amounts of D_2_R-like binding sites. Therefore, in the present investigation, images of D_2_R-like binding were coregistered with morphological images obtained with a dedicated small animal MRI tomograph, which allowed the transference of the pre-defined cortical and subcortical VOIs of the Paxinos standard rat brain MRI (Schiffer et al., 2006) to the functional SPECT images and, thus, permitted the first-time *in vivo* imaging analysis of radioligand binding to D_2_R-like binding sites in relevant regions of the rat nigrostriatal and mesolimbic system such as NAC, THAL, SN/VTA, FC, MC, PC, aHIPP, and pHIPP. Additionally, the relation between D_2_R-like binding in these regions and motor/exploratory behaviors was assessed with both correlation analysis and cluster analysis.

## Materials and methods

### Animals

Imaging studies of D_2_R-like binding sites were conducted on 32 adult male Wistar rats (ZETT, Heinrich-Heine University, Düsseldorf, Germany), weighing 429 ± 42 g [mean ± standard deviation (SD)]. Thereby, animals underwent one SPECT measurement in BAS and one SPECT measurement plus behavioral testing after challenge with MUS or BIC (*n* = 16, respectively). Morphological images were obtained on eight further male Wistar rats (ZETT, Heinrich-Heine University, Düsseldorf, Germany; weight: 434 ± 37 g). Findings on striatal D_2_R-like binding and behavioral data were previously published, with binding data, however, having been obtained with a different mode of analysis (see above; Nikolaus et al., 2017). Rats were maintained in standard macrolon cages (590 × 380 × 200 mm; three animals per cage) in a climate cabinet (Scantainer, Scanbur BK, Karslunde, Denmark; temperature: 20°C; air humidity; 70%) with an artificial ligh-dark cycle (lights on at 6:00 a.m., lights off at 6:00 p.m.) and food and water freely available. The study was carried out in accordance with the recommendations of the “Principles of laboratory animal care” (NIH publication No. 86-23, revised 1985) and the German Law on the Protection of Animals. The protocol was approved by the regional authority (Landesamt für Natur, Umwelt und Verbraucherschutz, Nordland-Westfalen, Recklinghausen, Germany).

### SPECT studies

SPECT measurements of D_2_R-like binding sites were performed with the TierSPECT (Schramm et al., 2000) as previously described (Nikolaus et al., 2017). Briefly, each rat underwent imaging studies in BAS and after intraperitoneal (i.p.) injection of MUS (Sigma-Aldrich, Taufkirchen, Germany; dose: 1 mg/kg, concentration: 1 mg/ml) or BIC (Sigma-Aldrich, Taufkirchen, Germany; dose: 1 mg/kg, concentration: 1 mg/ml).

Subsequently, animals were anesthetized with ketaminehydrochloride (Ketavet®, Pharmacia GmbH, Erlangen, Germany; dose: 0.9 ml/kg i.p., concentration: 100 mg/ml) and xylazinehydrochloride (Rompun® Bayer, Leverkusen, Germany; dose: 0.4 ml/kg i.p., concentration: 0.02 mg/ml). The employed radioligand [^123^I]S-3-iodo-N-(1-ethyl-2-pyrrolidinyl) methyl-2-hydroxy-6-methoxy benzamide ([^123^I]IBZM) has a high affinity for binding sites of the D_2_R-like subtype [D_2_R: inhibition constant (*K*_*i*_) = 1.6 nM, D_3_R: *K*_*i*_ = 2.2 nM; (Videbaek et al., 2000)]. [^123^I]IBZM (GE Healthcare, München, Germany; activity: 25.4 ± 3.6 MBq, concentration: 3.4 × 10^−9^ g/ml, specific activity: >74 TBq/mmol at reference time) was injected into the lateral tail vein. SPECT measurements were started 75 min after pharmacological challenge and 45 min after radioligand administration. Data were acquired over 60 min; thus, animals were kept under anesthesia for a total of 105 min. As to the selection of doses and time intervals between application of challenges, radioligand injection, and initiation of behavioral and SPECT measurements the reader is referred to a previous publication (Nikolaus et al., 2017).

### MRI studies

Upon anesthization with ketaminehydrochloride (dose: 0.45 ml/kg i.p., concentration: 100 mg/ml) and xylazinehydrochloride (dose: 0.2 ml/kg i.p., concentration: 0.02 mg/ml), rat heads were scanned with a dedicated small animal MRI tomograph (MRS3000 Pre-clinical MRI, 3.0 T, MR Solutions, Guildford, UK). A rat body volume coil with an inner diameter of 54 mm was used to transmit the radio frequency pulses and to receive the MR signals. A standard gradient echo pilot scan in three orthogonal directions was used for the positioning of a 3D fast low angle shot (FLASH) MR sequence to acquire high-resolution anatomical images (Haase et al., 1986). The measurement parameters for the 3D FLASH sequence were as follows: image matrix: 192 × 192 × 96, interpolated by zero-filling before reconstrution to a 256 × 256 × 128 matrix in coronal slice orientation; field of view: 64 × 64 × 44 mm; spatial resolution: 0.25 × 0.25 × 0.69 mm; repetition time: 30 ms; echo time: 4.87 ms; excitation flip angle: 30°; total acquisition time: 553 s.

### Behavioral studies

Rats underwent behavioral measurements in an open field (Phenotyper®, Noldus Information Technology, Wageningen, The Netherlands; dimensions: 45 × 45 × 56 cm) as previously described (Nikolaus et al., 2013, 2014a, 2016, 2017). Briefly, traveled distance (cm) as well as durations (s) and frequencies (*n*) of motor and exploratory behaviors (ambulation, sitting, rearing, head-shoulder motility, grooming) were registered in blocks of 5 min for a total of 30 min using EthoVision XT (Noldus Information Technology, Wageningen, The Netherlands).

### Evaluation of SPECT imaging studies

D_2/3_R imaging data were evaluated with PMOD (version 3.5, PMOD Technologies Ltd., Zürich, Switzerland). Firstly, the SPECT image of each rat was coregistered with a MR image of an animal of the same weight. Then, the respective MR image was coregistered with the Paxinos standard rat brain MRI (Schiffer et al., 2006) provided by PMOD. The necessary mathematical transformations were saved. The SPECT image as coregistered with the fitting MRI was imported using these transformations, which allowed creation of an overlay with the Paxinos standard rat brain MRI. On these overlays, the following volumes of interest (VOIs) were defined: CP, NAC, THAL, SN/VTA, FC, MC, PC, aHIPP, and pHIPP. The tomographic resolution of the employed SPECT camera amounts to 2.8 and 3.4 mm for ^99m^Tc and ^123^I, respectively (Schramm et al., 2000). According to the rat brain atlas, all these regions have maximum craniocaudal (CC) and one-sided mediolateral (ML) and dorsoventral (DV; vertical or oblique) dimensions in the range of or beyond the spatial resolution of the imaging system: CP, CC: >4.5 mm, ML: ~3.4 mm, DV: ~5 mm; NAC, CC: >3 mm, ML: ~2.5 mm, DV: ~3 mm; THAL, CC: >6.0 mm, ML: ~4.5 mm, DV: ~3.5 mm; SN/VTA, CC: >2.5 mm, ML: ~2.8 mm, DV: ~3.2 mm; FC, CC: >3.4 mm, ML: ~3.1 mm, DV: ~3.8 mm; MC, CC: >8.4 mm, ML: ~4.0 mm, DV: ~4.2 mm; PC, CC: >7.8 mm, ML: ~3.0 mm, DV: ~8.0 mm; HIPP, CC: >8 mm, ML: ~5.0 mm, DV: ~6.5 mm (Paxinos and Watson, 2014). Moreover, in autoradiographic studies performed with a variety of radioligands including [^123^I]IBZM, these regions have been shown to express D_2_R-like binding sites (Bouthenet et al., 1987; Verhoeff et al., 1991). Since [^123^I]IBZM accumulation in the cerebellum (CER) is non-specific, the CER was used as reference region (REF; see Supplementary Figure). Estimations of regional binding potentials (BPs) for BAS and challenges were obtained according to the simplified reference tissue model by computing ratios of radioactvity counts obtained in the specifically-bound compartments (CP, NAC, THAL, SN/VTA, FC, MC, PC, aHIPP, and pHIPP) to radioactivity counts in the CER (Ichise et al., 2001).

### Statistical analysis

Distributions of both regional BPs and behavioral data were tested for normality with the non-parametric Kolmogorov–Smirnov test (α = 0.05). Neither in BAS, nor after MUS or BIC, regional BPs were uniformly normally distributed (0.021 ≤ *p* ≤ 0.20). This also held for behavioral parameters after both MUS and BIC (0.0001 ≤ *p* ≤ 0.20).

Medians with 25-/75-percentiles were computed for regional BPs. Regional BPs were compared between pre-treatment conditions (BAS_MUS_ vs. MUS, BAS_BIC_ vs. BIC) with the Wilcoxon signed rank test for paired samples (two-tailed, α = 0.05). Moreover, percentual differences of BPs relative to BAS were computed for both MUS and BIC. No corrections of the alpha value were made for multiple comparisons. Calculations were performed with IBM SPSS Statistics 23 (IBM SPSS Software Germany, Ehningen, Germany).

In order to gauge the extent of association between regional radioligand binding and motor/exploratory parameters, Spearman rank correlation coefficients (*r*; α = 0.05) were computed for the individual BPs and behavioral data in the individual time frames (1–5, 6–10, 11–15, 16–20, 21–25, and 26–30 min). Calculations were performed with SigmaStat (version 3.5, Systat Software Inc., Erkrath, Germany).

In addition, cluster analyses were performed for regional BPs and behavioral data (traveled distance and durations and frequencies of ambulation, sitting, rearing, head-shoulder motility, and grooming) in the individual time frames (1–5, 6–10, 11–15, 16–20, 21–25, and 26–30 min) after MUS and after BIC. The individual data were standardized using the Z-transformation [(individual value – variable mean)/SD from the variable mean]. The number of clusters and the centroid mean of each variable in them were determined with an X-means algorithm (Pelleg and Moore, 2000). Cluster analyses were calculated with Rapid Miner (version 5.3., Rapid-I GmbH, Dortmund, Germany). The individual values of the behavioral parameters, which entered correlation analysis and cluster analysis, are given for each animal in the Supplementary Table.

## Results

### D_2/3_R binding

**Figure 1:**
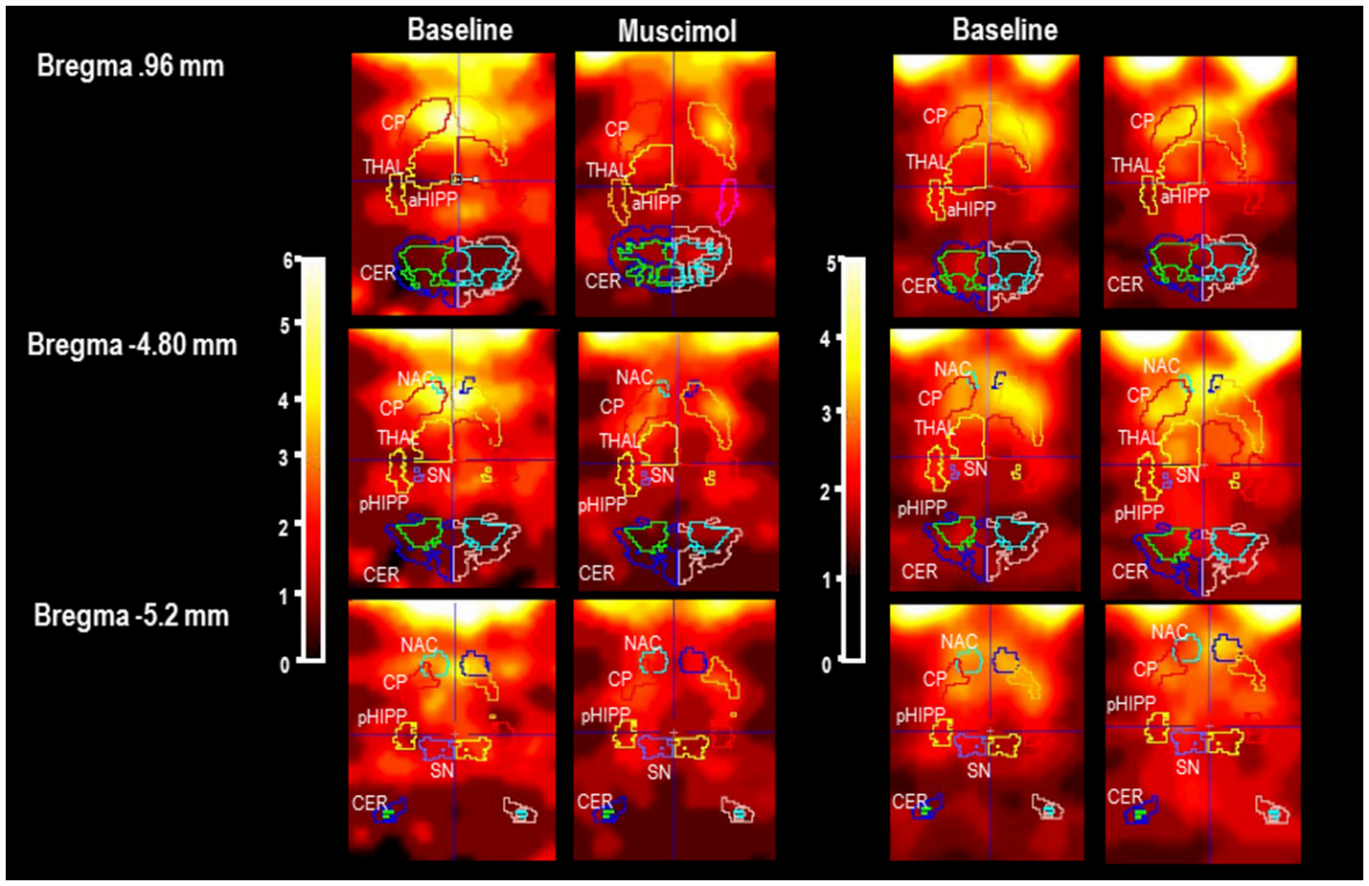
Individual slices at different positions from Bregma (Paxinos and Watson, 2014) in baseline and after pharmacological challenge with 1 mg/kg muscimaol or 1 mg/kg bicuculline (BAS_MUS_ and MUS, BAS_BIC_ and BIC). CP, caudateputamen; NAC, nucleus accumbens; SN, substantia nigra; VTA, ventral tegmental area; THAL, thalamus; PFC, prefrontal cortex; MC, motor cortex; PC, parietal cortex; aHIPP, anterior hippocamus; pHIPP, posterior hippocampus; CER, cerebellum. In the present example, the rat treated with MUS shows a reduction of [^123^I]IBZM accumulation in CP, NAC, THAL, and SN/VTA. The rat treated with BIC shows a slight elevation of [^123^I]IBZM accumulation in CP and THAL.

In Figure 1, characteristic coronal images of regional [^123^I]IBZM accumulations in the BAS condition and after challenge with MUS and BIC, respectively, are presented at different positions from Bregma (Paxinos and Watson, 2014). SPECT images in the conditions BAS_MUS_ and MUS were obtained in the same rat. This also holds for BAS_BIC_ and BIC.

**Figure 2:**
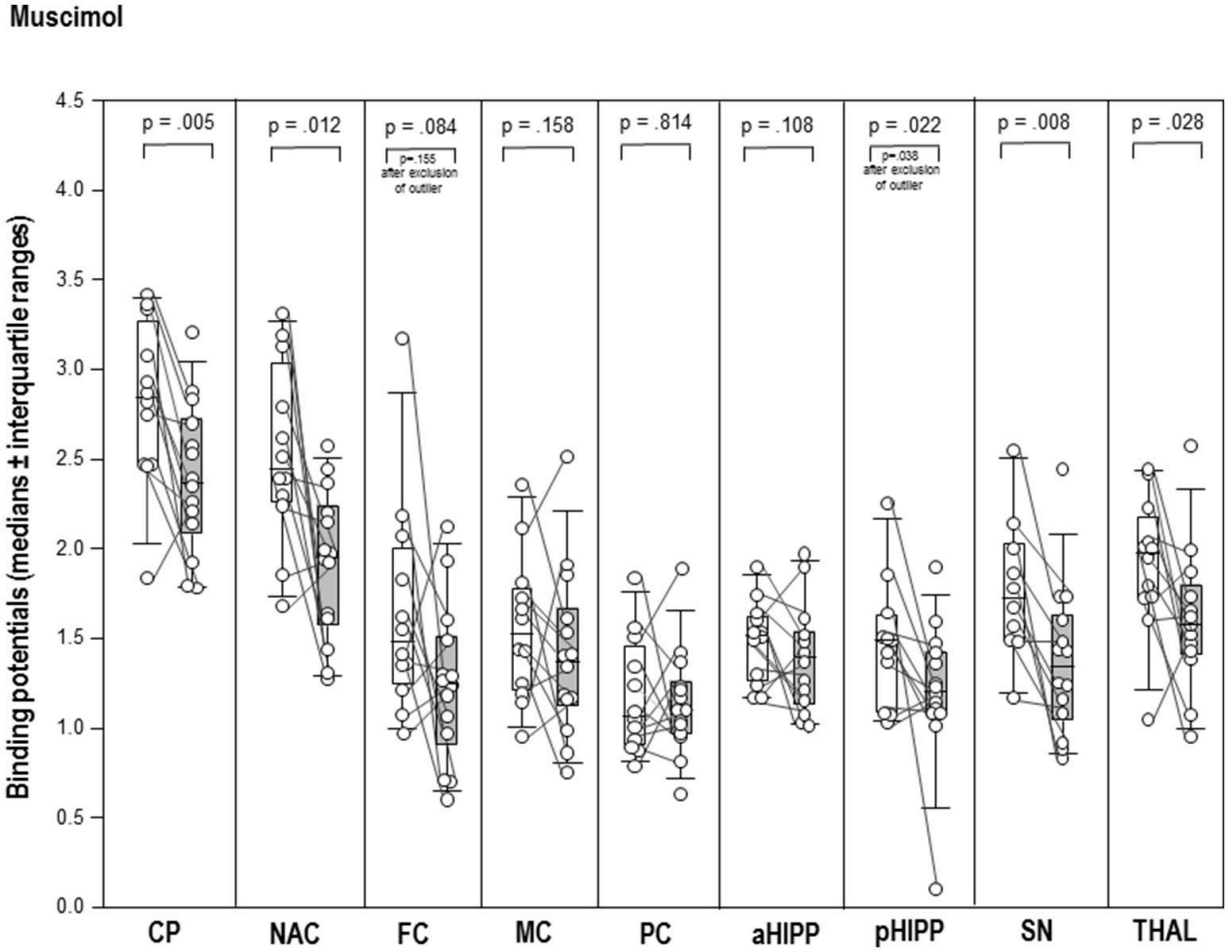
Binding potentials in baseline (white) and after challenge with 1 mg/kg muscimol (gray). Rendered are medians and 25-/75- (boxes) and 9-/95-quartiles (whiskers). The circles represent the individual animals. For significant between-group differences, the respective *p*-values are given (Wilcoxon signed rank test for paired samples, two-tailed, α = 0.05).

After MUS (Figure 2), BPs were significantly reduced in CP (−17%, *p* = 0.005), NAC (−20%, *p* = 0.012), pHIPP (−19%, *p* = 0.022), SN/VTA (−22%, *p* = 0.008), and THAL (−20%, *p* = 0.028) relative to BAS_MUS_. There were no differences between BAS_MUS_ and MUS in FC, MC, PC, aHIPP, and CER (0.108 ≤ *p* ≤ 0.433).

**Figure 3:**
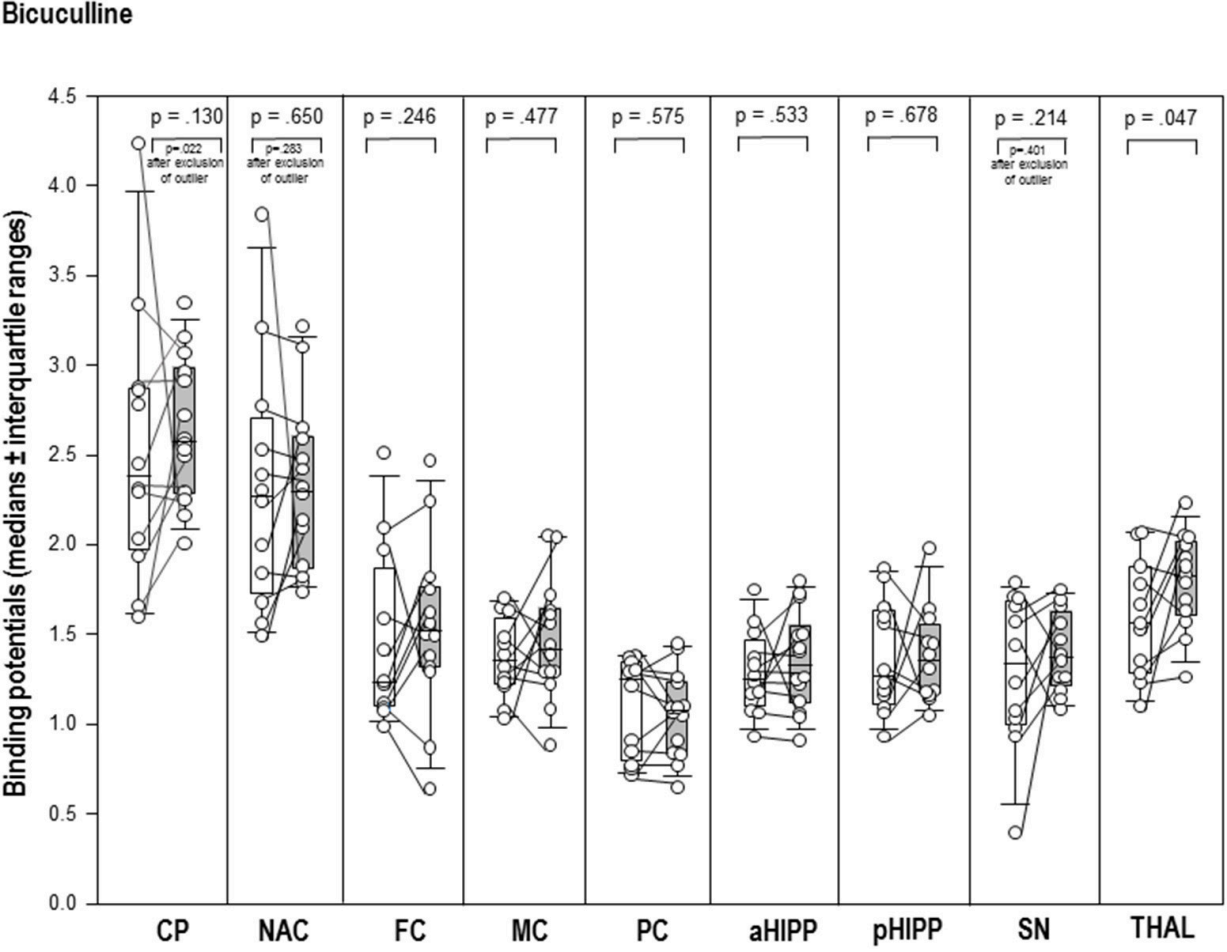
Binding potentials in baseline (white) and after challenge with 1 mg/kg bicuculline (gray). Rendered are medians and 25-/75- (boxes) and 9-/95-quartiles (whiskers). The circles represent the individual animals. For significant between-group differences, the respective *p*-values are given (Wilcoxon signed rank test for paired samples, two-tailed, α = 0.05).

After BIC (Figure 3), the thalamic BP was significantly elevated (+17%, *p* = 0.047) compared to BAS_BIC_. Likewise, the post-challenge BP in the CP was significantly increased relative to the BP in BAS (+8%, *p* = 0.022; Figure 3). There were no differences between BAS_BIC_ and BIC in NAC, SN/VTA, FC, MC, PC, aHIPP, pHIPP, and CER (0.130 ≤ *p* ≤ 0.678).

### Correlations between regional D_2/3_R binding and behaviors

Table 1 shows the significant (0.0001 ≤ *p* ≤ 0.05) positive and negative correlations between regional BPs and behavioral parameters in the individual time frames after treatment with MUS or BIC.

**Table 1:** Significant positive and negative correlations (0.0001 ≤ *p* ≤ 0.051) between D_2/3_R binding in CP, caudateputamen; NAC, nucleus accumbens; THAL, thalamus; SN/VTA, substantia nigra/ventral tegmental area; FC, frontal cortex; MC, motor cortex; PC, parietal cortex; aHIPP, anterodorsal hippocampus; pHIPP, posterior hippocampus; and behavioral parameters in the individual time frames after challenge with 1 mg/kg muscimol (MUS) or 1 mg/kg bicuculline (BIC).

**Behavioral parameter**	**CP**		**NAC**		**THAL**		**SN/VTA**		**FC**		**MC**		**PC**		**aHIPP**		**pHIPP**	
	**MUS**	**BIC**	**MUS**	**BIC**	**MUS**	**BIC**	**MUS**	**BIC**	**MUS**	**BIC**	**MUS**	**BIC**	**MUS**	**BIC**	**MUS**	**BIC**	**MUS**	**BIC**
Traveled distance min 1–5 min 6–10 min 11–15 min 16–20 min 21–25 min 26–30	0.552 0.556				0.610		0.658											0.580
Ambulation duration min 1–5 min 6–10 min 11–15 min 16–20 min 21–25 min 26–30	0.560						0.623											0.606
Ambulation frequency min 1–5 min 6–10 min 11–15 min 16–20 min 21–25 min 26–30	0.564		0.523				0.642											0.638
Sitting duration min 1–5 min 6–10 min 11–15 min 16–20 min 21–25 min 26–30				−0.574		−0.644	0.579				0.543	−0.646						
Sitting frequency min 1–5 min 6–10 min 11–15 min 16–20 min 21–25 min 26–30	−0.587		−0.656 −0.60		−0.697	0.644		0.557				−0.661	0.663		0.581			
Rearing duration min 1–5 min 6–10 min 11–15 min 16–20 min 21–25 min 26–30			0.542				0.559			0.529						0.576		0.569
Rearing frequency min 1–5 min 6–10 min 11–15 min 16–20 min 21–25 min 26–30					0.689		0.674			0.665			−0.549			0.568		0.569
Duration of head-shoulder motility min 1–5 min 6–10 min 11–15 min 16–20 min 21–25 min 26–30			0.534				−0.583				0.565							0.727
Frequency of head-shoulder motility																		
min 1–5	−0.538			−0.776												−0.583	−0.543	
min 6–10																		
min 11–15																		
min 16–20	0.521		0.676															
min 21–25																		
min 26–30	0.561						0.626		0.738		0.703						0.546	0.643
Grooming duration min 1–5 min 6–10 min 11–15 min 16–20 min 21–25 min 26–30			−0.619	0.525					−0.575								−0.524	
Grooming frequency min 1–5 min 6–10 min 11–15 min 16–20 min 21–25 min 26–30	−0.722		−0.740								−0.534				−0.616		−0.714	

*Positive and negative correlations are marked in red and blue, respectively*.

After MUS, lower D_2/3_R binding in CP, NAC, SN/VTA, FC, MC, aHIPP, and pHIPP was associated with a reduction of motor/exploratory parameters in the second half of the testing session and an increase of sitting and grooming primarily between 11 and 15 min.

For BIC, the correlation analysis showed an association between lower D_2/3_R binding in CP, NAC, THAL, MC, and aHIPP and more sitting and head-shoulder motility as well as less traveled distance and rearing primarily during the first 5 min of the testing session.

### Cluster analysis

#### MUS

The cluster analysis (Table 2) of regional D_2/3_R binding data and behavioral variables in the individual time frames yielded two clusters (cluster 1: *n* = 7, cluster 2: *n* = 9). Relative to the second cluster, the first one was characterized by lower centroid means of D_2/3_R binding in CP, NAC, THAL, and FC and higher centroid means of D_2/3_R binding in SN/VTA, MC, PC, aHIPP, and pHIPP. Moreover, relative to the second cluster, centroid means in the first one were lower for (1) sitting duration and frequency in the fifth time frame, (2) traveled distance, ambulation duration, ambulation frequency, and frequency of head-shoulder motility in the first, second, third, fourth, and sixth time frame, and (3) rearing duration, rearing frequency, and duration of head-shoulder motility in all time frames. In contrast, centroid means in the first cluster were higher for (1) traveled distance, ambulation duration, ambulation frequency, and frequency of head-shoulder motility in the fifth time frame, (2) sitting duration and sitting freuency in the first, second, third, fourth, and sixth time frame, and (3) both grooming duration and grooming frequency in all time frames.

**Table 2:** Centroid means of D_2/3_R binding in CP, caudateputamen; NAC, nucleus accumbens; THAL, thalamus; SN/VTA, substantia nigra/ventral tegmental area; FC, frontal cortex; MC, motor cortex; PC, parietal cortex; aHIPP, anterodorsal hippocampus; pHIPP, posterior hippocampus; as well as traveled distance and ambulation and frequency of ambulation, sitting, rearing, head-shoulder motility, and grooming in the individual time frames (min 1–5, 6–10, 11–15, 16–20, 21–25, and 26–30) in rats pre-treated with 1 mg/kg muscimol (MUS) or 1 mg/kg biculline (BIC) after Z-transformation of the individual data.

		**MUS**		**BIC**	
**Variable**	**Time frame**	**C1**	**C2**	**C1**	**C2**
D_2/3_R-CP		−0.143	0.184	−0.335	0.201
D_2/3_R-NAC		−0.298	0.384	−0.270	0.162
D_2/3_R-THAL		−0.045	0.058	−0.082	0.049
D_2/3_R-SN/VTA		0.012	−0.015	0.552	−0.331
D_2/3_R-FC		−0.035	0.046	0.143	−0.086
D_2/3_R-MC		0.034	−0.045	−0.226	0.136
D_2/3_R-PC		0.123	−0.158	0.437	−0.262
D_2/3_R-aHIPP		0.021	−0.027	0.444	−0.267
D_2/3_R-pHIPP		0.103	−0.132	0.694	−0.416
Traveled distance	min 1–5	−0.510	0.656	0.318	−0.191
Ambulation duration	min 1–5	−0.429	0.552	0.183	−110
Ambulation frequency	min 1–5	−0.490	0.630	0.260	−0.156
Sitting duration	min 1–5	0.486	−0.624	−0.332	0.199
Sitting frequency	min 1–5	0.516	−0.664	−0.060	0.036
Rearing duration	min 1–5	−0.576	0.741	0.547	−0.328
Rearing frequency	min 1–5	−0.551	0.709	0.650	−0.390
Head-shoulder motility duration	min 1–5	−0.203	0.261	−0.214	0.129
Head-shoulder motility frequency	min 1–5	−0.281	0.362	0.067	−0.004
Grooming duration	min 1–5	0.071	−0.091	−0.246	0.148
Grooming frequency	min 1–5	0.175	−0.225	−0.070	0.042
Traveled distance	min 6–10	−0.539	0.693	0.431	−0.259
Ambulation duration	min 6–10	−0.420	0.540	0.509	−0.305
Ambulation frequency	min 6–10	−0.562	0.723	0.456	−0.273
Sitting duration	min 6–10	0.538	−0.692	−0.117	0.070
Sitting frequency	min 6–10	0.510	−0.656	0.223	−0.134
Rearing duration	min 6–10	−0.546	0.702	0.043	−0.026
Rearing frequency	min 6–10	−0.563	0.724	0.250	−0.150
Head-shoulder motility duration	min 6–10	−0.559	0.719	0.384	−0.231
Head-shoulder motility frequency	min 6–10	−0.590	0.758	0.642	−0.385
Grooming duration	min 6–10	0.278	−0.358	−0.332	0.199
Grooming frequency	min 6–10	0.045	−0.058	−0.323	0.194
Traveled distance	min 11–15	−0.278	0.358	0.783	−0.470
Ambulation duration	min 11–15	−0.273	0.351	0.727	−0.436
Ambulation frequency	min 11–15	−0.332	0.426	0.900	−0.540
Sitting duration	min 11–15	0.374	−0.481	0.106	−0.064
Sitting frequency	min 11–15	0.131	−0.168	0.504	−0.303
Rearing duration	min 11–15	−0.426	0.547	0.467	−0.280
Rearing frequency	min 11–15	−0.234	0.300	0.621	−0.372
Head-shoulder motility duration	min 11–15	−0.632	0.812	0.782	−0.469
Head-shoulder motility frequency	min 11–15	−0.522	0.671	1.014	−0.608
Grooming duration	min 11–15	0.398	−0.512	−0.826	0.496
Grooming frequency	min 11–15	0.155	−0.199	−0.499	0.299
Traveled distance	min 16–20	−0.294	0.378	0.299	−0.179
Ambulation duration	min 16–20	−0.350	0.450	0.305	−0.183
Ambulation frequency	min 16–20	−0.340	0.437	0.257	−0.154
Sitting duration	min 16–20	0.277	−0.356	−0.386	0.232
Sitting frequency	min 16–20	0.266	−0.342	−0.066	0.039
Rearing duration	min 16–20	−0.408	0.525	0.058	−0.035
Rearing frequency	min 16–20	−0.183	0.235	0.085	−0.051
Head-shoulder motility duration	min 16–20	−0.224	0.288	0.342	−0.205
Head-shoulder motility frequency	min 16–20	−0.225	0.290	0.309	−0.186
Grooming duration	min 16–20	0.005	−0.007	0.281	−0.168
Grooming frequency	min 16–20	0.103	−0.132	0.628	−0.377
Traveled distance	min 21–25	0.172	−0.221	0.412	−0.259
Ambulation duration	min 21–25	0.245	−0.315	0.582	−0.349
Ambulation frequency	min 21–25	0.185	−0.238	0.412	−0.247
Sitting duration	min 21–25	−0.148	0.187	−0.622	0.373
Sitting frequency	min 21–25	−0.146	0.187	0.049	−0.029
Rearing duration	min 21–25	−0.016	0.020	0.356	−0.214
Rearing frequency	min 21–25	−0.040	0.051	0.380	−0.229
Head-shoulder motility duration	min 21–25	−0.045	0.058	0.297	−0.178
Head-shoulder motility frequency	min 21–25	0.189	−0.243	0.382	−0.229
Grooming duration	min 21–25	0.219	−0.281	0.475	−0.285
Grooming frequency	min 21–25	0.276	−0.354	0.525	−0.315
Traveled distance	min 26–30	−0.359	0.461	1.054	−0.633
Ambulation duration	min 26–30	−0.329	0.423	1.070	−0.642
Ambulation frequency	min 26–30	−0.336	0.432	0.964	−0.578
Sitting duration	min 26–30	0.216	−0.277	−0.857	0.514
Sitting frequency	min 26–30	0.033	0.461	0.486	−0.291
Rearing duration	min 26–30	−0.376	0.483	0.850	−0.510
Rearing frequency	min 26–30	−0.361	0.464	0.922	−0.553
Head-shoulder motility duration	min 26–30	−0.347	0.446	0.880	−0.528
Head-shoulder motility frequency	min 26–30	−0.288	0.371	0.984	−0.590
Grooming duration	min 26–30	0.364	−0.468	0.489	−0.294
Grooming frequency	min 26–30	0.015	−0.019	0.523	−0.314

#### BIC

For treatment with BIC, the cluster analysis (Table 2) of regional D_2/3_R binding data and behavioral variables in the individual time frames also yielded two clusters (cluster 1: *n* = 6, cluster 2: *n* = 10). Relative to the second cluster, the first one was characterized by lower centroid means of D_2/3_R binding in CP, NAC, THAL, and MC and higher centroid means of D_2/3_R binding in SN/VTA, FC, aHIPP, and pHIPP. Moreover, relative to the second cluster, centroid means of the first one were lower for (1) sitting frequency in the first and fourth time frame, (2) duration of head shoulder motility in the first and second time frame, and (3) sitting duration in the first, second, fourth, fifth, and sixth time frame, but higher for (1) sitting duration in the third time frame, (2) sitting frequency in the second, third, fifth, and sixth time frame, (3) grooming duration and grooming frequency in the fourth, fifth, and sixth time frame, (4) traveled distance, ambulation duration, and ambulation frequency, duration of head-shoulder motility in the second, third, fourth, fifth, and sixth time frame, and (5) rearing duration, rearing frequency, and frequency of head-shoulder motility in all time frames.

## Discussion

### D_2/3_R binding

Pre-treatment with the GABA_A_R agonist MUS in a dose of 1 mg/kg significantly reduced D_2/3_R binding in CP (−17%), NAC (−20%), pHIPP (−19%), SN/VTA (−22%), and THAL (−20%). In contrast, the GABA_A_R antagonist BIC in a dose of 1 mg/k increased D_2/3_R binding in CP (+8%) and THAL (+17%).

Former investigations have shown that MUS injections into VTA (Klitenick et al., 1992) and NAC (Yoshida et al., 1997; Aono et al., 2008) elevated DA concentrations in these regions. Moreover, MUS increased neostriatal and intranigral DA levels when applied into the SN (Santiago and Westerink, 1992) and into the GP (Cobb and Abercrombie, 2002, 2003). In a precedent study, we had presented first evidence that MUS also after systemic application elevated neostriatal DA levels, leading to a competition between endogenous DA and the exogenous radioligand and a subsequent reduction of radioligand binding to the neostriatal D_2_R-like subtype (Nikolaus et al., 2017). The present analysis of these imaging data, using MRI overlays additionally yielded evidence of diminished D_2/3_R binding in NAC, THAL, SN/VTA, and pHIPP, reflecting increased availability of DA also in these regions.

Former *in vivo* microdialysis studies have shown elevated DA levels in CP (Smolders et al., 1995 and NAC (Yan, 1999; Aono et al., 2008) upon administration of BIC into these regions. Furthermore, BIC augmented DA concentrations in the CP, when injected into PFC (Karreman and Moghaddam, 1996; Matsumoto et al., 2003) and SN (Santiago and Westerink, 1992; Westerink et al., 1992) and in the NAC, when injected into the VTA (Ikemoto et al., 1997). This is contrasted by the results of our *in vivo* imaging study, which revealed increased D_2/3_R binding in CP and THAL upon systemic challenge with BIC, indicating decrements of synaptic DA in these regions. This disagreement, firstly, may be due to the applied dose, which, in the present study (2.7 mM) was considerably higher than in the precedent investigations (10–100 μM; Santiago and Westerink, 1992; Westerink et al., 1992; Smolders et al., 1995; Karreman and Moghaddam, 1996; Ikemoto et al., 1997; Yan, 1999; Matsumoto et al., 2003; Aono et al., 2008). A second reason may be the fact, that in the present study the pharmacological challenges were administered systemically in contrast to the localized injection in the other investigations. It may be inferred that the simultaneous targeting of all regions expressing GABA_A_R binding sites may have influenced the GABAergic (and DAergic) afferents and efferents, regulating nigral, tegmental, striatal, thalamic, and cortical function differently from the localized approach in the *in vivo* microdialysis studies.

In the present study, systemic application of the GABA_A_R agonist MUS reduced D_2/3_R binding in striatum (CP, NAC), THAL, SN/VTA, and pHIPP, reflecting increased availability of DA in these regions. The D_2_R has two interconvertible binding states for DA, which are referred to as high-affinity (G-protein-coupled) and low-affinity (G-protein-uncoupled) state (De Lean et al., 1982). To our knowledge, the individual affinities of [^123^I]IBZM for the high- and the low-affinity D_2_R configuration have not yet been determined. However, studies on [^11^C]raclopride—a further D_2_R antagonistic benzamide analog widely used for the assessment of D_2_R binding and competion with endogenous DA (Laruelle, 2000)—have shown similar affinities for both types of D_2_Rs (Seneca et al., 2006). From this may be inferred that also [^123^I]IBZM binds to D_2_R-like binding sites in both configurations and that the regional BPs obtained in the present investigation may be considered to reflect the regional densities of D_2/3_Rs as such, irrespective of the individual contributions of either affinity state. This not only holds for the BPs in BAS, but also for BPs obtained after challenge with either MUS or BIC, and exempts us from the necessity to differentiate between the individual effects exerted by D_2/3_Rs in the high- and in the low-affinity configuration. With this simplification in mind, the following actions may be hypothesized to occur in the individual brain regions: firstly, within the neostriatal microcircuits (Groves, 1983), the GABA_A_R agonist action of MUS can be assumed to lead to a decline of DA efflux. In the DAergic system, DA concentrations undergo regulation by autoreceptors of the D_2_R subtype, which are situated at the presynaptic terminal (Langer, 1974). Consequently, the decrement of striatal DA efflux elicited by MUS is likely to diminish DA binding to presynaptic D_2_ autoreceptors, leading to a reduction of feedback inhibition, subsequent elevation of DA efflux and the observed reduction of radioligand binding to the D_2/3_R in the CP.

The increased GABA_A_R agonistic action in the CP can be inferred to augment the inhibition of the SN via the direct pathway. The same effect is exerted by GABAergic thalamonigral and DAergic striatonigral efferents (the latter via inhibitory D_2/3_R binding sites). The consequence of both GABAergic and DAergic inhibition is the decrease of nigral DA levels and the subsequent reduction of inhibitory D_2_ autoreceptor action. Via the indirect pathway, the SN is disinhibited, which—together with the increased input of excitatory striatonigral efferents (via D_1_R binding sites) and the mentioned decline of D_2_ autoreceptor action—can be hypothesized to cause the net increase of nigral DA reflected by the reduction of D_2/3_R under the present experimental conditions.

The increased GABA_A_R agonistic action in the indirect pathway disinhibits the THAL. Furthermore, elevated DAergic input from the CP both disinhibits (via D_2/3_ heteroreceptors) and inhibits (via D_1_ heteroreceptors) the THAL in the direct and indirect pathway, respectively (Bolam et al., 2000). As indicated by the observed reduction of thalamic D_2/3_R binding, these effects altogether appear to result in a net increase of thalamic DA.

From the THAL, GLUergic efferents run to the neocortex, which in turn sends GLUergic projections back to the CP (Rouse et al., 2000). Thalamic excitation of the neocortex can be expected to enhance the excitatory input exerted by the descending corticostriatal fibers. The stimulation of striatal N-methyl-D-aspartate (NMDA) receptors (Clow and Jhamandas, 1989) likely joins the action of both nigral and striatal D_2_ autoreceptors in augmenting striatal DA levels in response to the GABA_A_R agonist action of MUS.

In line with the action of MUS on the CP, leading to an elevated inhibition of the SN, the action of MUS on the NAC may be conceived to enhance the inhibition of the VTA, resulting in a reduction of DA release in the NAC. Again, the diminished DA efflux can be hypothesized to decrease DA binding to presynaptic D_2_ autoreceptors, leading to a decrease of feedback inhibition, subsequent elevation of DA efflux and the observed reduction of D_2/3_R binding in the NAC.

Moreover, elevated GABA_A_R agonist action in the NAC can be assumed to increase the inhibition of the GP, leading to disinhibition of the THAL. This likely adds to the increased GABAergic and DAergic action in the indirect and direct pathways, respectively, causing a net increase of thalamic DA and the observed reduction of thalamic D_2/3_R binding.

The NAC receives inhibitory GABAergic and DAergic efferents from the PFC (Lee et al., 2014) and the THAL (Hara et al., 1989), respectively. The increase of DA in the THAL probably acts jointly with the inhibition of VTA and NAC to reduce DA levels in the latter region, leading to the decrease of feedback inhibition and subsequent elevation of DA efflux hypothesized to underlie the observed decline of D_2/3_R binding.

The HIPP receives DAergic neurons originating in the VTA (Nazari-Serenjeh et al., 2011). Hence, the MUS-induced inhibition of the VTA is likely to diminish the DA release in the HIPP, again resulting in reduced feedback inhibition, subsequent enhancement of DA release, and the observed decline of radioligand binding to the D_2/3_R in the pHIPP. The HIPP sends GLUergic efferents to the NAC (Nazari-Serenjeh et al., 2011), with the stimulation of NMDA receptors likely joining the action of D_2_ autoreceptors in augmenting DA levels in the NAC and subsequently also in the THAL.

In the present study, systemic application of the GABA_A_R agonist BIC increased D_2/3_R binding in CP and THAL, reflecting decreased availability of DA in these regions. Firstly, it may be hypothesized that, within the microcircuits in the CP, the GABA_A_R antagonistic action elicited an increase of DA efflux, which is compensated by an enhancement of feedback inhibition and subsequent reduction of DA levels, as indicated in the present study by the elevation of D_2/3_R binding in the CP.

*In vivo* imaging studies did not show alterations of D_2/3_R binding (and DA) in SN/VTA and NAC. Therefore, actions of BIC on thalamic DA via the NAC can be dismissed. It may be inferred, however, that the increased GABA_A_R antagonistic action in the indirect pathway leads to an inhibition of the THAL. Furthermore, also the BIC-induced reduction of DAergic input from the CP decreases both thalamic inhibition (via D_2/3_ heteroreceptors) and excitation (via D_1_ heteroreceptors) in the direct and indirect pathway, respectively. Altogether, these effects may be assumed to result in a net decrease of thalamic DA and the observed elevation of thalamic D_2/3_R binding.

### Rat behavior

Imaging and behavioral data obtained after MUS can be grouped into two clusters with the first one characterized by lower D_2/3_R binding in CP, NAC, THAL, and FC and higher D_2/3_R binding in SN/VTA, MC, PC, aHIPP, and pHIPP and the second one displaying the opposite. Moreover, the animals of the first cluster showed less motor/exploratory behaviors and more sitting and grooming, whereas the animals of the second cluster exhibited more motor/exploratory activity and less sitting and grooming. Hence, the cluster analysis implies an inverse relationship between DA levels in the nigrostriatal/mesolimbic system and motor/exploratory activity after GABA_A_R agonistic treatment. In addition, correlation analysis revealed that, after MUS, lower D_2/3_R binding (and higher DA) throughout the entire DAergic system (CP, NAC, SN/VTA, FC, MC, aHIPP, and pHIPP) was associated with a reduction of motor/exploratory parameters in the second half of the testing time and an increase of sitting and grooming primarily from 11 to 15 min. From this, we infer that, after systemic MUS, DA concentrations in the nigrostriatal and mesolimbic system started to rise around 11 min post-injection. Furthermore, the reductions of D_2/3_R binding in the imaging studies indicate that the synaptic DA levels were still elevated in 75–135 min after MUS challenge.

For imaging and behavioral data obtained after BIC, the cluster analysis yielded two clusters with the first one characterized by lower D_2/3_R binding in CP, NAC, THAL, and MC and higher D_2/3_R binding in SN/VTA, FC, aHIPP, and pHIPP and the second one displaying the opposite. Moreover, the animals of the first cluster showed less sitting, but more motor/exploratory behaviors as well as grooming throughout the testing time, whereas those of the second cluster exhibited more sitting and less motor/exploratory activity and grooming. Thus, interestingly, after GABA_A_R antagonistic treatment, leading to decreased DA in CP and THAL but normal DA in the other brain regions, a direct relationship emerges between DA concentration in the nigrostriatal/mesolimbic system and motor/exploratory activity.

Yet, the correlation analysis revealed an association between lower D_2/3_R binding (and higher DA) throughout the nigrostriatal/mesolimbic system (CP, NAC, THAL, MC, and aHIPP) and more sitting and head-shoulder motility as well as less traveled distance, and rearing primarily during the first 5 min of testing time. This, for one, indicates that the GABA_A_R antagonist elicited an almost immediate decline of synaptic DA concentrations, which, in NAC and THAL, was still visible at the time of *in vivo* imaging studies, and which is reflected by the immediate reduction of motor/exploratory behaviors. Secondly, however, it seems that the association between nigrostriatal/mesolimbic DA and motor/exploratory behaviors is different for generally high (as after L-DOPA Nikolaus et al., 2016 or MUS) and generally low or normal levels of DA (as after BIC) with the former inversely and the latter directly related to motor/exploratory activity. Further *in vivo* imaging studies of D_2/3_R binding after increasing doses of L-DOPA, MUS, and BIC are required to gain more information on this regulation mechanism, which is likely to involve tight region-specific D_2_ autoreceptor action.

### Appraisal

Against the present findings the objection can be raised that D_2/3_R SPECT and MR images were not obtained on the same rats, which may have led to misalignments not only between D_2/3_R SPECT and MRI of individual animals, but also between D_2/3_R SPECT and the standard Paxinos rat brain MRI. However, the BPs obtained for the CP in the present study (BAS_MUS_: 2.845, MUS: 2.37) were in agreement with the previous mean neostriatal equilibrium ratios (V_3_“) of 1.818 (BAS_MUS_) and 1.397 (MUS), corresponding to BPs of 2.818 and 2.397, respectively (Nikolaus et al., 2017). Also after BIC, the BPs obtained for the CP (BAS_BIC_: 2.380, BIC: 2.575) were consistent with the fromer findings (BAS_BIC_: V_3_“ = 1.532, BP = 2.532; BIC: V_3_“ = 1.646, BP = 2.646; Nikolaus et al., 2017), which argues in favor of the present method. Although also the previous data had indicated an increase of neostriatal D_2/3_R binding after BIC, this difference had not reached statistical significance. It may be argued, thus, that the MRI-based mode of analysis is superior to the former method of defining a small VOI around the neostriatal hot spot of maximum radioactivity accumulation, since it allows the definition of exact anatomical VOIs within the striatum. Besides, also the definition of VOIs other than striatal ones is rendered possible, resulting in the present first-time evidence of GABAergic effects on thalamic, nigral/ventral tegmental, and hippocampal DA obtained with *in vivo* imaging methods.

The maximum VOI diameters are either in the range or beyond the spatial resolution of the employed imaging system. It must be borne in mind, however, that the quantification of D_2/3_R binding in those portions of VOIs, which fall short of the full width at half maximum, may be hampered by partial volume effects, leading to underestimations of radioligand accumulations. Another pitfall may be overestimation of radioligand binding due to spill-over from regions with high radioligand accumulation such as the extraorbital Harderian glands to the adjacent VOIs of FC, CP, and NAC, or from the CP to NAC, THAL, and aHIPP. It can be maintained, however, that (semi)quantitative data—also of regions such as NAC, THAL, SN/VTA, and HIPP—are comparable between BAS and challenge in the present study as well as between investigations on other rats perfomed with the same imaging tool (Nikolaus et al., 2011, 2013, 2014a, 2016, 2017). As soon as further imaging studies on D_2_R-like binding sites in these regions will be available, it will be interesting to see, in which range the present binding ratios lie in comparison to binding ratios obtained with new-generation SPECT systems.

Dysfunctions of GABA_A_Rs and D_2_R-like binding sites have been implied in neuropsychiatric disorders including anxiety disorders and schizophrenia (Nikolaus et al., 2010, 2014b,c). Interestingly, the decline of GABA_A_R function in anxiety disorders involves the whole nigrostriatal and mesolimbic system, while the function of D_2_R-like binding sites is merely impaired in the CP. Contrarily, in schizophrenia, the reduction of GABA_A_R binding is confined to the neocortex, while D_2_R-like binding sites are dysfunctional throughout the nigrostriatal and mesolimbic system. This implies that the emotional and behavioral changes characteristic for the individual diseases are related to regional neurochemic alterations of receptor function. The present study in the rat constitutes an important step toward unraveling the complex interdependencies of behaviors and the neurochemistry of DA and GABA using a two-modality *in vivo* imaging approach.

## Conclusion

Findings indicate direct GABAergic control over synaptic DA levels in the migrostriatal and mesolimbic system in relation to behavioral action. The may be of relevance for neuropsychiatric conditions such as anxiety disorder and schizophrenia, which are characterized by both DAergic and GABAergic dysfunction.

## Author contributions

SN, MADSS, JPH, CA, GA and H-WM: Experimental design; SN, H-JW, AM-L and FW: Performance of imaging and behavioral studies; SN and MB: Evaluation and statistical analysis; SN, MB, CA, MADSS, JPH, HH, H-WM: Interpreation of findings; SN, H-JW, CA, MADSS, JPH, GA, HH, H-WM: Writing and editing of the manuscript.

### Conflict of interest statement

The authors declare that the research was conducted in the absence of any commercial or financial relationships that could be construed as a potential conflict of interest.

## Supplementary material

Supplementary Figure VOI definition on overlays of SPECT images of [^123^I]IBZM binding to the D_2_R (which had been formerly coregistered with the MRI of a rat of the same weight), with the standard Paxinos rat brein MRI (Schiffer et al., 2006). CP, caudateputamen; NAC, nucleus accumbens; SN, substantia nigra; VTA, THAL, thalamus; PFC, prefrontal cortex; FC, frontal cortex; MC, motor cortex; PC, parietal cortex; aHIPP, anterior hippocamus; pHIPP, posterior hippocampus; CER, cerebellum.

Supplementary Table Behavioral data (either the number [n] of behaviors or the time [s], the animals engaged in a specific behavior) obtained for the individual rats after challenge with either 1 mg/kg muscimol (MUS) or 1 mg/kg bicuculline (BIC) in the individual time frames.
