# Supplementary figures and images for: GABAergic Control of Nigrostriatal and Mesolimbic Dopamine in the Rat Brain
Source: Front Behav Neurosci. 2018 Mar 14;12:38. doi: 10.3389/fnbeh.2018.00038 (PMC5862131; doi:10.3389/fnbeh.2018.00038)

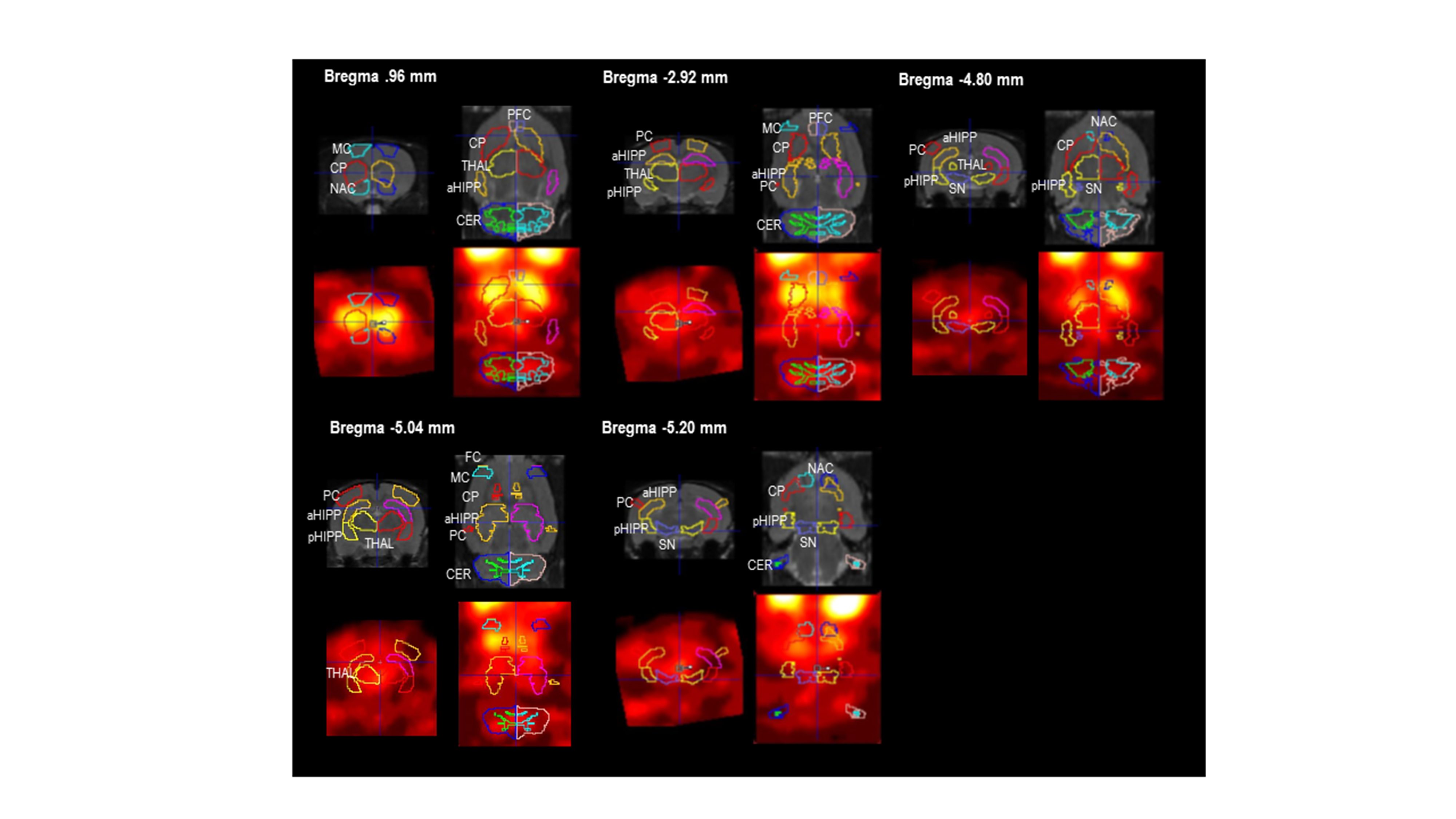

Supplement: Supplementary Figure — VOI definition on overlays of SPECT images of [123I]IBZM binding to the D2R (which had been formerly coregistered with the MRI of a rat of the same weight), with the standard Paxinos rat brein MRI (Schiffer et al., 2006). CP, caudateputamen; NAC, nucleus accumbens; SN, substantia nigra; VTA, THAL, thalamus; PFC, prefrontal cortex; FC, frontal cortex; MC, motor cortex; PC, parietal cortex; aHIPP, anterior hippocamus; pHIPP, posterior hippocampus; CER, cerebellum. [file Image1.TIF]
