# Supplementary material for: GABAergic Control of Nigrostriatal and Mesolimbic Dopamine in the Rat Brain
Source: Front Behav Neurosci. 2018 Mar 14;12:38. doi: 10.3389/fnbeh.2018.00038 (PMC5862131; doi:10.3389/fnbeh.2018.00038)
Supplement: Supplementary Table — Behavioral data (either the number [n] of behaviors or the time [s], the animals engaged in a specific behavior) obtained for the individual rats after challenge with either 1 mg/kg muscimol (MUS) or 1 mg/kg bicuculline (BIC) in the individual time frames. [file Table1.DOC]

Supplmentary table. Behavioral data (either the number [n] of behaviors or the time [s], the animals engaged in a specific behavior) obtained for the individual rats after challenge with either 1 mg/kg muscimol (MUS) or 1 mg/kg bicuculline (BIC) in the individual time frames.

A. 1-5 min

| Rat | Treatment | Traveled  distance (cm)  1-5 min | Ambulation  duration (s)  1-5 min | Ambulation  frequency (n)  1-5 min | Sitting  duration (s)  1-5 min | Sitting  frequency (n)  1-5 min | Rearing  duration (s)  1-5 min | Rearing  frequency (n)  1-5 min | Head-shoulder  motility duration (s)  1-5 min | Head-shoulder  motility frequency (n)  1-5 min | Grooming  duration (s)  1-5 min | Grooming  frequency (n)  1-5 min |
| --- | --- | --- | --- | --- | --- | --- | --- | --- | --- | --- | --- | --- |
| 1 | MUS | 4454.43 | 129.00 | 68.00 | 0.00 | 0.00 | 102.00 | 55.00 | 67.40 | 35.00 | 1.60 | 1.00 |
| 2 | MUS | 2689.81 | 87.00 | 33.00 | 7.00 | 2.00 | 42.00 | 23.00 | 113.80 | 31.00 | 50.20 | 2.00 |
| 3 | MUS | 3903.56 | 127.60 | 57.00 | 0.00 | 0.00 | 62.20 | 35.00 | 102.00 | 46.00 | 8.20 | 4.00 |
| 4 | MUS | 3048.12 | 95.80 | 50.00 | 0.00 | 0.00 | 65.60 | 29.00 | 138.60 | 42.00 | 0.00 | 0.00 |
| 5 | MUS | 2683.69 | 94.80 | 34.00 | 40.80 | 12.00 | 38.40 | 14.00 | 99.60 | 32.00 | 26.40 | 3.00 |
| 6 | MUS | 2137.01 | 84.00 | 33.00 | 13.20 | 5.00 | 24.40 | 11.00 | 97.00 | 38.00 | 81.40 | 8.00 |
| 7 | MUS | 3152.32 | 94.20 | 43.00 | 5.00 | 1.00 | 41.40 | 19.00 | 143.00 | 45.00 | 16.40 | 3.00 |
| 8 | MUS | 1134.25 | 38.20 | 12.00 | 150.40 | 11.00 | 22.20 | 8.00 | 73.80 | 19.00 | 15.40 | 1.00 |
| 9 | MUS | 4350.67 | 113.00 | 48.00 | 0.00 | 0.00 | 68.40 | 33.00 | 104.40 | 45.00 | 14.20 | 2.00 |
| 10 | MUS | 3798.97 | 116.40 | 54.00 | 15.00 | 6.00 | 50.00 | 23.00 | 113.60 | 49.00 | 5.00 | 1.00 |
| 11 | MUS | 3661.58 | 113.00 | 57.00 | 0.60 | 1.00 | 66.60 | 37.00 | 93.60 | 40.00 | 26.20 | 5.00 |
| 12 | MUS | 2174.61 | 85.60 | 26.00 | 100.80 | 5.00 | 26.40 | 10.00 | 87.20 | 32.00 | 0.00 | 0.00 |
| 13. | MUS | 1318.11 | 41.00 | 18.00 | 102.00 | 7.00 | 26.00 | 7.00 | 116.00 | 26.00 | 15.00 | 2.00 |
| 14 | MUS | 3637.95 | 98.40 | 49.00 | 1.80 | 2.00 | 51.40 | 26.00 | 128.40 | 52.00 | 20.00 | 1.00 |
| 15 | MUS | 3733.99 | 102.80 | 54.00 | 24.80 | 8.00 | 50.20 | 28.00 | 103.40 | 53.00 | 18.80 | 2.00 |
| 16 | MUS | 1949.77 | 62.60 | 21.00 | 168.60 | 10.00 | 15.20 | 7.00 | 53.60 | 31.00 | 0.00 | 0.00 |
| 17 | BIC | 5092.02 | 93.80 | 48.00 | 0.00 | 0.00 | 86.60 | 50.00 | 116.40 | 48.00 | 3.20 | 1.00 |
| 18 | BIC | 2085.64 | 62.20 | 28.00 | 92.20 | 18.00 | 31.20 | 12.00 | 114.40 | 42.00 | 0.00 | 0.00 |
| 19 | BIC | 3547.30 | 116.40 | 56.00 | 7.20 | 4.00 | 53.20 | 27.00 | 123.20 | 61.00 | 0.00 | 0.00 |
| 20 | BIC | 3898.39 | 110.80 | 52.00 | 7.60 | 6.00 | 58.60 | 29.00 | 115.60 | 56.00 | 7.40 | 2.00 |
| 21 | BIC | 3198.52 | 91.80 | 50.00 | 0.00 | 0.00 | 67.40 | 38.00 | 128.20 | 49.00 | 12.60 | 2.00 |
| 22 | BIC | 4644.07 | 120.60 | 53.00 | 0.00 | 0.00 | 85.60 | 31.00 | 84.80 | 41.00 | 9.00 | 1.00 |
| 23 | BIC | 4777.69 | 133.00 | 64.00 | 0.00 | 0.00 | 61.80 | 34.00 | 89.20 | 50.00 | 16.00 | 4.00 |
| 24 | BIC | 4675.46 | 99.20 | 56.00 | 0.00 | 0.00 | 79.80 | 40.00 | 115.40 | 50.00 | 5.60 | 2.00 |
| 25 | BIC | 3069.67 | 89.20 | 43.00 | 11.60 | 2.00 | 34.40 | 18.00 | 135.40 | 39.00 | 29.40 | 2.00 |
| 26 | BIC | 4026.11 | 91.60 | 61.00 | 0.00 | 0.00 | 74.20 | 35.00 | 121.40 | 65.00 | 12.80 | 3.00 |
| 27 | BIC | 3967.77 | 112.00 | 65.00 | 2.00 | 1.00 | 45.40 | 30.00 | 114.20 | 64.00 | 26.40 | 7.00 |
| 28 | BIC | 3998.75 | 99.20 | 64.00 | 1.00 | 1.00 | 90.60 | 45.00 | 97.00 | 56.00 | 12.20 | 3.00 |
| 29 | BIC | 4565.71 | 120.20 | 71.00 | 0.00 | 0.00 | 49.60 | 38.00 | 126.60 | 68.00 | 3.60 | 1.00 |
| 30 | BIC | 6726.21 | 129.80 | 85.00 | 11.00 | 6.00 | 73.60 | 51.00 | 75.80 | 56.00 | 9.80 | 4.00 |
| 31 | BIC | 3851.42 | 94.40 | 51.00 | 18.40 | 3.00 | 49.60 | 29.00 | 135.60 | 56.00 | 2.00 | 1.00 |
| 32 | BIC | 3102.19 | 94.20 | 46.00 | 54.20 | 9.00 | 30.20 | 15.00 | 117.00 | 59.00 | 4.40 | 1.00 |

B. 6-10 min

| Rat | Treatment | Traveled  distance (cm)  6-10 min | Ambulation  duration (s)  6-10 min | Ambulation  frequency (n)  6-10 min | Sitting  duration (s)  6-10 min | Sitting  frequency (n)  6-10 min | Rearing  duration (s)  6-10 min | Rearing  frequency (n)  6-10 min | Head-shoulder  motility duration (s)  6-10 min | Head-shoulder  motility frequency (n)  6-10 min | Grooming  duration (s)  6-10 min | Grooming  frequency (n)  6-10 min |
| --- | --- | --- | --- | --- | --- | --- | --- | --- | --- | --- | --- | --- |
| 1 | MUS | 2562.88 | 82.20 | 35.00 | 3.60 | 2.00 | 76.20 | 31.00 | 97.00 | 33.00 | 41.00 | 5.00 |
| 2 | MUS | 1463.95 | 65.80 | 23.00 | 32.20 | 4.00 | 18.20 | 8.00 | 173.00 | 31.00 | 10.80 | 3.00 |
| 3 | MUS | 1617.08 | 66.60 | 28.00 | 70.40 | 12.00 | 18.80 | 10.00 | 125.80 | 36.00 | 18.40 | 3.00 |
| 4 | MUS | 2330.22 | 69.40 | 31.00 | 27.00 | 4.00 | 40.40 | 19.00 | 150.20 | 42.00 | 13.00 | 3.00 |
| 5 | MUS | 1087.97 | 38.80 | 14.00 | 69.20 | 13.00 | 17.40 | 5.00 | 96.60 | 25.00 | 78.00 | 5.00 |
| 6 | MUS | 1666.35 | 77.60 | 19.00 | 43.40 | 5.00 | 18.60 | 6.00 | 61.60 | 21.00 | 98.80 | 7.00 |
| 7 | MUS | 1211.63 | 51.00 | 18.00 | 2.00 | 1.00 | 1.60 | 1.00 | 166.20 | 23.00 | 79.20 | 4.00 |
| 8 | MUS | 117.69 | 0.00 | 0.00 | 244.80 | 7.00 | 0.00 | 0.00 | 26.40 | 6.00 | 28.80 | 1.00 |
| 9 | MUS | 2507.39 | 92.00 | 29.00 | 0.00 | 0.00 | 32.60 | 12.00 | 146.60 | 36.00 | 28.80 | 4.00 |
| 10 | MUS | 2171.35 | 68.80 | 30.00 | 31.00 | 6.00 | 44.80 | 16.00 | 155.40 | 43.00 | 0.00 | 0.00 |
| 11 | MUS | 1986.61 | 70.80 | 33.00 | 3.20 | 1.00 | 29.20 | 14.00 | 143.20 | 34.00 | 53.60 | 5.00 |
| 12 | MUS | 1592.22 | 94.80 | 33.00 | 81.40 | 3.00 | 28.60 | 17.00 | 95.20 | 22.00 | 0.00 | 0.00 |
| 13. | MUS | 642.75 | 22.00 | 8.00 | 173.00 | 13.00 | 16.80 | 3.00 | 62.20 | 23.00 | 26.00 | 5.00 |
| 14 | MUS | 2720.68 | 72.60 | 39.00 | 11.60 | 3.00 | 58.40 | 23.00 | 156.00 | 49.00 | 1.40 | 1.00 |
| 15 | MUS | 1488.34 | 44.40 | 16.00 | 105.20 | 7.00 | 33.40 | 10.00 | 77.20 | 28.00 | 39.80 | 4.00 |
| 16 | MUS | 2587.87 | 59.60 | 24.00 | 110.60 | 10.00 | 31.00 | 10.00 | 98.80 | 34.00 | 0.00 | 0.00 |
| 17 | BIC | 3709.73 | 96.20 | 44.00 | 0.00 | 0.00 | 73.20 | 27.00 | 122.60 | 38.00 | 8.00 | 2.00 |
| 18 | BIC | 1113.25 | 42.40 | 18.00 | 158.80 | 17.00 | 3.60 | 1.00 | 85.60 | 34.00 | 9.60 | 1.00 |
| 19 | BIC | 2287.87 | 95.20 | 38.00 | 29.40 | 11.00 | 33.60 | 18.00 | 128.40 | 51.00 | 13.40 | 2.00 |
| 20 | BIC | 3229.02 | 100.00 | 42.00 | 21.80 | 7.00 | 46.20 | 17.00 | 111.00 | 47.00 | 21.00 | 2.00 |
| 21 | BIC | 1516.57 | 48.00 | 21.00 | 2.00 | 1.00 | 31.00 | 11.00 | 95.80 | 28.00 | 123.20 | 4.00 |
| 22 | BIC | 1145.42 | 29.60 | 13.00 | 34.80 | 7.00 | 23.80 | 5.00 | 76.20 | 25.00 | 135.60 | 8.00 |
| 23 | BIC | 2351.63 | 73.60 | 27.00 | 8.20 | 2.00 | 41.40 | 15.00 | 77.40 | 32.00 | 99.40 | 10.00 |
| 24 | BIC | 3222.90 | 92.40 | 50.00 | 0.00 | 0.00 | 76.80 | 34.00 | 106.40 | 49.00 | 24.40 | 1.00 |
| 25 | BIC | 2593.58 | 85.80 | 43.00 | 9.00 | 3.00 | 33.80 | 11.00 | 120.80 | 42.00 | 50.60 | 2.00 |
| 26 | BIC | 2632.97 | 74.40 | 35.00 | 0.00 | 0.00 | 55.40 | 15.00 | 128.00 | 39.00 | 42.20 | 2.00 |
| 27 | BIC | 1792.98 | 53.60 | 28.00 | 1.00 | 1.00 | 16.60 | 7.00 | 116.60 | 35.00 | 112.20 | 6.00 |
| 28 | BIC | 2595.18 | 70.40 | 48.00 | 3.00 | 2.00 | 57.60 | 32.00 | 111.60 | 62.00 | 57.40 | 4.00 |
| 29 | BIC | 2721.71 | 66.60 | 40.00 | 1.40 | 1.00 | 52.80 | 20.00 | 104.00 | 35.00 | 75.20 | 3.00 |
| 30 | BIC | 3261.78 | 83.80 | 50.00 | 53.60 | 13.00 | 24.60 | 15.00 | 100.80 | 52.00 | 37.20 | 2.00 |
| 31 | BIC | 2794.70 | 77.00 | 41.00 | 9.20 | 1.00 | 73.60 | 22.00 | 117.20 | 33.00 | 23.00 | 2.00 |
| 32 | BIC | 2038.94 | 57.80 | 24.00 | 35.00 | 6.00 | 51.20 | 31.00 | 67.00 | 38.00 | 89.00 | 5.00 |

C. 11-15 min

| Rat | Treatment | Traveled  distance (cm)  11-15 min | Ambulation  duration (s)  11-15 min | Ambulation  frequency (n)  11-15 min | Sitting  duration (s)  11-15 min | Sitting  frequency (n)  11-15 min | Rearing  duration (s)  11-15 min | Rearing  frequency (n)  11-15 min | Head-shoulder  motility duration (s)  11-15 min | Head-shoulder  motility frequency (n)  11-15 min | Grooming  duration (s)  11-15 min | Grooming  frequency (n)  11-15 min |
| --- | --- | --- | --- | --- | --- | --- | --- | --- | --- | --- | --- | --- |
| 1 | MUS | 790.11 | 21.60 | 10.00 | 79.00 | 5.00 | 17.60 | 6.00 | 179.00 | 18.00 | 2.80 | 1.00 |
| 2 | MUS | 857.29 | 39.00 | 15.00 | 102.60 | 10.00 | 29.40 | 6.00 | 129.00 | 28.00 | 0.00 | 0.00 |
| 3 | MUS | 206.57 | 4.80 | 1.00 | 44.40 | 6.00 | 0.00 | 0.00 | 57.40 | 9.00 | 193.40 | 4.00 |
| 4 | MUS | 847.58 | 32.60 | 17.00 | 76.00 | 6.00 | 9.20 | 3.00 | 166.00 | 27.00 | 16.20 | 2.00 |
| 5 | MUS | 158.12 | 0.00 | 0.00 | 164.40 | 9.00 | 0.00 | 0.00 | 22.00 | 6.00 | 113.60 | 3.00 |
| 6 | MUS | 1051.19 | 34.20 | 12.00 | 41.60 | 4.00 | 12.20 | 4.00 | 89.20 | 15.00 | 122.80 | 4.00 |
| 7 | MUS | 1075.10 | 54.20 | 19.00 | 43.60 | 4.00 | 1.60 | 1.00 | 146.00 | 21.00 | 54.60 | 4.00 |
| 8 | MUS | 155.51 | 0.00 | 0.00 | 196.80 | 11.00 | 0.00 | 0.00 | 18.60 | 6.00 | 84.60 | 4.00 |
| 9 | MUS | 1842.37 | 57.60 | 19.00 | 12.40 | 2.00 | 31.00 | 6.00 | 144.60 | 25.00 | 54.40 | 3.00 |
| 10 | MUS | 1774.65 | 80.60 | 24.00 | 31.60 | 7.00 | 29.40 | 11.00 | 131.80 | 36.00 | 26.60 | 1.00 |
| 11 | MUS | 714.13 | 29.20 | 10.00 | 127.20 | 10.00 | 0.00 | 0.00 | 109.20 | 22.00 | 34.40 | 5.00 |
| 12 | MUS | 739.96 | 43.00 | 17.00 | 141.20 | 7.00 | 9.60 | 7.00 | 65.60 | 26.00 | 40.60 | 3.00 |
| 13. | MUS | 2120.41 | 81.40 | 28.00 | 61.60 | 1.00 | 31.40 | 11.00 | 125.60 | 28.00 | 0.00 | 0.00 |
| 14 | MUS | 1390.56 | 56.80 | 27.00 | 7.20 | 3.00 | 32.20 | 9.00 | 163.20 | 34.00 | 40.60 | 4.00 |
| 15 | MUS | 1048.49 | 30.20 | 13.00 | 161.80 | 7.00 | 15.20 | 8.00 | 39.60 | 14.00 | 53.20 | 4.00 |
| 16 | MUS | 668.41 | 19.00 | 6.00 | 206.20 | 17.00 | 10.40 | 3.00 | 64.40 | 21.00 | 0.00 | 0.00 |
| 17 | BIC | 2857.93 | 80.20 | 38.00 | 0.00 | 0.00 | 60.60 | 21.00 | 141.00 | 40.00 | 18.20 | 4.00 |
| 18 | BIC | 756.73 | 25.40 | 8.00 | 165.60 | 18.00 | 8.00 | 2.00 | 83.60 | 26.00 | 17.40 | 2.00 |
| 19 | BIC | 1501.89 | 48.40 | 26.00 | 42.00 | 12.00 | 28.60 | 9.00 | 178.20 | 41.00 | 2.80 | 1.00 |
| 20 | BIC | 1634.61 | 53.20 | 20.00 | 58.60 | 18.00 | 8.40 | 4.00 | 94.60 | 36.00 | 85.20 | 8.00 |
| 21 | BIC | 2379.73 | 55.00 | 30.00 | 4.20 | 3.00 | 43.60 | 12.00 | 174.20 | 41.00 | 23.00 | 1.00 |
| 22 | BIC | 1423.52 | 46.20 | 16.00 | 7.60 | 3.00 | 32.20 | 5.00 | 84.40 | 23.00 | 129.60 | 6.00 |
| 23 | BIC | 1069.40 | 39.00 | 11.00 | 9.60 | 3.00 | 2.60 | 1.00 | 25.80 | 15.00 | 223.00 | 7.00 |
| 24 | BIC | 726.56 | 21.40 | 8.00 | 0.00 | 0.00 | 2.40 | 2.00 | 43.00 | 16.00 | 233.20 | 10.00 |
| 25 | BIC | 550.17 | 2.00 | 2.00 | 45.80 | 4.00 | 4.60 | 2.00 | 17.20 | 9.00 | 230.40 | 3.00 |
| 26 | BIC | 967.27 | 26.20 | 11.00 | 47.60 | 6.00 | 7.00 | 4.00 | 75.20 | 17.00 | 144.00 | 4.00 |
| 27 | BIC | 1998.54 | 52.80 | 28.00 | 8.60 | 5.00 | 31.40 | 8.00 | 96.80 | 39.00 | 110.40 | 11.00 |
| 28 | BIC | 1700.88 | 28.20 | 20.00 | 49.80 | 13.00 | 45.60 | 20.00 | 105.40 | 46.00 | 71.00 | 4.00 |
| 29 | BIC | 1479.90 | 27.00 | 13.00 | 2.80 | 1.00 | 40.20 | 13.00 | 37.00 | 16.00 | 193.00 | 5.00 |
| 30 | BIC | 1987.99 | 77.00 | 40.00 | 98.20 | 14.00 | 12.40 | 5.00 | 72.20 | 39.00 | 40.20 | 1.00 |
| 31 | BIC | 1581.30 | 47.60 | 18.00 | 20.20 | 5.00 | 19.20 | 6.00 | 131.40 | 23.00 | 81.60 | 4.00 |
| 32 | BIC | 1781.54 | 46.40 | 19.00 | 39.20 | 6.00 | 44.40 | 13.00 | 80.80 | 29.00 | 89.20 | 4.00 |

D. 16-20 min

| Rat | Treatment | Traveled  distance (cm)  16-20 min | Ambulation  duration (s)  16-20 min | Ambulation  frequency (n)  16-20 min | Sitting  duration (s)  16-20 min | Sitting  frequency (n)  16-20 min | Rearing  duration (s)  16-20 min | Rearing  frequency (n)  16-20 min | Head-shoulder  motility duration (s)  16-20 min | Head-shoulder  motility frequency (n)  16-20 min | Grooming  duration (s)  16-20 min | Grooming  frequency (n)  16-20 min |
| --- | --- | --- | --- | --- | --- | --- | --- | --- | --- | --- | --- | --- |
| 1 | MUS | 904.33 | 37.40 | 12.00 | 113.80 | 7.00 | 16.20 | 5.00 | 106.80 | 24.00 | 25.80 | 3.00 |
| 2 | MUS | 1060.09 | 66.00 | 19.00 | 14.40 | 1.00 | 5.40 | 1.00 | 168.60 | 22.00 | 45.60 | 2.00 |
| 3 | MUS | 134.51 | 0.00 | 0.00 | 219.20 | 5.00 | 0.00 | 0.00 | 13.80 | 2.00 | 67.00 | 7.00 |
| 4 | MUS | 149.10 | 2.20 | 2.00 | 212.20 | 11.00 | 0.00 | 0.00 | 77.00 | 14.00 | 8.60 | 1.00 |
| 5 | MUS | 1149.16 | 46.40 | 14.00 | 166.80 | 7.00 | 6.60 | 3.00 | 80.20 | 17.00 | 0.00 | 0.00 |
| 6 | MUS | 730.45 | 22.40 | 9.00 | 53.20 | 6.00 | 5.40 | 2.00 | 63.00 | 16.00 | 156.00 | 5.00 |
| 7 | MUS | 530.03 | 31.60 | 11.00 | 146.40 | 9.00 | 0.00 | 0.00 | 122.00 | 20.00 | 0.00 | 0.00 |
| 8 | MUS | 164.20 | 0.00 | 0.00 | 221.00 | 6.00 | 0.00 | 0.00 | 12.20 | 3.00 | 66.80 | 2.00 |
| 9 | MUS | 1616.78 | 62.60 | 18.00 | 20.60 | 5.00 | 23.60 | 4.00 | 144.80 | 24.00 | 48.40 | 2.00 |
| 10 | MUS | 1055.87 | 59.00 | 16.00 | 39.00 | 8.00 | 34.40 | 2.00 | 99.00 | 21.00 | 68.60 | 3.00 |
| 11 | MUS | 728.02 | 33.40 | 12.00 | 205.80 | 4.00 | 11.60 | 4.00 | 35.80 | 14.00 | 13.40 | 3.00 |
| 12 | MUS | 778.97 | 40.20 | 11.00 | 76.20 | 6.00 | 13.20 | 5.00 | 82.00 | 20.00 | 88.40 | 6.00 |
| 13. | MUS | 976.44 | 37.00 | 16.00 | 31.80 | 8.00 | 5.20 | 2.00 | 164.00 | 24.00 | 62.00 | 1.00 |
| 14 | MUS | 602.18 | 16.60 | 9.00 | 11.00 | 4.00 | 2.80 | 1.00 | 53.80 | 13.00 | 215.80 | 3.00 |
| 15 | MUS | 650.93 | 16.00 | 7.00 | 150.00 | 7.00 | 8.80 | 3.00 | 40.80 | 11.00 | 84.40 | 2.00 |
| 16 | MUS | 235.91 | 2.00 | 1.00 | 188.20 | 20.00 | 0.00 | 0.00 | 80.40 | 24.00 | 29.40 | 3.00 |
| 17 | BIC | 2535.15 | 58.20 | 27.00 | 0.00 | 0.00 | 63.80 | 18.00 | 107.80 | 33.00 | 70.20 | 6.00 |
| 18 | BIC | 116.92 | 0.00 | 0.00 | 265.60 | 16.00 | 0.00 | 0.00 | 34.40 | 16.00 | 0.00 | 0.00 |
| 19 | BIC | 1457.84 | 48.20 | 18.00 | 19.20 | 5.00 | 23.00 | 8.00 | 92.60 | 27.00 | 117.00 | 6.00 |
| 20 | BIC | 1532.23 | 36.40 | 17.00 | 27.20 | 7.00 | 29.60 | 6.00 | 64.20 | 30.00 | 142.60 | 12.00 |
| 21 | BIC | 479.01 | 21.60 | 7.00 | 60.00 | 5.00 | 0.00 | 0.00 | 59.00 | 13.00 | 159.40 | 6.00 |
| 22 | BIC | 1203.43 | 41.20 | 14.00 | 126.60 | 12.00 | 36.80 | 8.00 | 93.00 | 29.00 | 2.40 | 2.00 |
| 23 | BIC | 1232.77 | 38.40 | 16.00 | 25.00 | 4.00 | 23.40 | 7.00 | 39.60 | 23.00 | 173.60 | 6.00 |
| 24 | BIC | 75.69 | 0.00 | 0.00 | 271.40 | 5.00 | 0.00 | 0.00 | 16.20 | 5.00 | 12.40 | 1.00 |
| 25 | BIC | 1678.20 | 49.00 | 26.00 | 107.40 | 9.00 | 26.60 | 6.00 | 109.60 | 37.00 | 7.40 | 1.00 |
| 26 | BIC | 90.50 | 0.00 | 0.00 | 215.40 | 11.00 | 0.00 | 0.00 | 30.00 | 8.00 | 54.60 | 4.00 |
| 27 | BIC | 933.08 | 19.80 | 10.00 | 4.20 | 3.00 | 4.00 | 1.00 | 31.20 | 16.00 | 240.80 | 5.00 |
| 28 | BIC | 325.27 | 0.00 | 0.00 | 134.60 | 11.00 | 0.00 | 0.00 | 49.20 | 14.00 | 116.20 | 5.00 |
| 29 | BIC | 1511.53 | 27.60 | 15.00 | 1.00 | 1.00 | 30.60 | 5.00 | 45.20 | 21.00 | 195.60 | 9.00 |
| 30 | BIC | 1344.64 | 39.40 | 20.00 | 153.00 | 10.00 | 11.20 | 5.00 | 38.00 | 26.00 | 58.40 | 3.00 |
| 31 | BIC | 95.93 | 0.00 | 0.00 | 291.40 | 4.00 | 0.00 | 0.00 | 8.60 | 4.00 | 0.00 | 0.00 |
| 32 | BIC | 2135.27 | 57.40 | 26.00 | 3.20 | 3.00 | 70.20 | 25.00 | 96.40 | 29.00 | 72.80 | 2.00 |

E. 21-25 min

| Rat | Treatment | Traveled  distance (cm)  21-25 min | Ambulation  duration (s)  21-25 min | Ambulation  frequency (n)  21-25 min | Sitting  duration (s)  21-25 min | Sitting  frequency (n)  21-25 min | Rearing  duration (s)  21-25 min | Rearing  frequency (n)  21-25 min | Head-shoulder  motility duration (s)  21-25 min | Head-shoulder  motility frequency (n)  21-25 min | Grooming  duration (s)  21-25 min | Grooming  frequency (n)  21-25 min |
| --- | --- | --- | --- | --- | --- | --- | --- | --- | --- | --- | --- | --- |
| 1 | MUS | 532.61 | 18.80 | 7.00 | 141.00 | 4.00 | 8.60 | 4.00 | 127.20 | 9.00 | 4.40 | 1.00 |
| 2 | MUS | 96.92 | 0.00 | 0.00 | 275.00 | 5.00 | 0.00 | 0.00 | 25.00 | 4.00 | 0.00 | 0.00 |
| 3 | MUS | 92.45 | 0.00 | 0.00 | 277.00 | 5.00 | 0.00 | 0.00 | 12.00 | 3.00 | 11.00 | 1.00 |
| 4 | MUS | 319.70 | 8.80 | 6.00 | 223.20 | 9.00 | 0.00 | 0.00 | 68.00 | 15.00 | 0.00 | 0.00 |
| 5 | MUS | 1135.97 | 73.00 | 17.00 | 16.40 | 2.00 | 0.00 | 0.00 | 99.40 | 21.00 | 111.20 | 4.00 |
| 6 | MUS | 963.91 | 42.00 | 13.00 | 83.00 | 2.00 | 12.80 | 4.00 | 83.20 | 19.00 | 79.00 | 2.00 |
| 7 | MUS | 1087.40 | 58.40 | 19.00 | 100.40 | 6.00 | 22.60 | 5.00 | 94.40 | 22.00 | 24.20 | 1.00 |
| 8 | MUS | 107.15 | 0.00 | 0.00 | 270.60 | 6.00 | 0.00 | 0.00 | 13.40 | 4.00 | 16.00 | 1.00 |
| 9 | MUS | 647.94 | 35.80 | 7.00 | 81.60 | 12.00 | 0.00 | 0.00 | 121.60 | 16.00 | 61.00 | 1.00 |
| 10 | MUS | 758.50 | 46.80 | 16.00 | 75.80 | 10.00 | 18.20 | 2.00 | 138.40 | 23.00 | 20.80 | 1.00 |
| 11 | MUS | 394.38 | 13.60 | 6.00 | 170.80 | 3.00 | 0.00 | 0.00 | 47.80 | 9.00 | 67.80 | 3.00 |
| 12 | MUS | 610.62 | 28.20 | 9.00 | 54.80 | 3.00 | 5.80 | 1.00 | 80.40 | 15.00 | 130.80 | 5.00 |
| 13. | MUS | 897.59 | 42.80 | 17.00 | 99.80 | 17.00 | 0.00 | 0.00 | 157.40 | 33.00 | 0.00 | 0.00 |
| 14 | MUS | 1428.98 | 33.40 | 18.00 | 135.60 | 11.00 | 32.80 | 11.00 | 82.80 | 28.00 | 15.40 | 2.00 |
| 15 | MUS | 2115.56 | 72.20 | 34.00 | 29.40 | 7.00 | 31.60 | 9.00 | 138.00 | 44.00 | 28.80 | 4.00 |
| 16 | MUS | 297.51 | 9.40 | 3.00 | 221.60 | 14.00 | 0.00 | 0.00 | 65.20 | 16.00 | 3.80 | 1.00 |
| 17 | BIC | 2377.86 | 69.80 | 28.00 | 1.60 | 1.00 | 34.40 | 15.00 | 122.20 | 37.00 | 72.00 | 5.00 |
| 18 | BIC | 450.98 | 14.40 | 6.00 | 179.60 | 28.00 | 2.40 | 1.00 | 103.60 | 33.00 | 0.00 | 0.00 |
| 19 | BIC | 1294.27 | 53.20 | 20.00 | 80.00 | 14.00 | 17.80 | 6.00 | 95.60 | 33.00 | 53.40 | 3.00 |
| 20 | BIC | 1480.08 | 53.20 | 19.00 | 59.20 | 14.00 | 22.20 | 7.00 | 79.00 | 29.00 | 86.40 | 6.00 |
| 21 | BIC | 1559.03 | 61.40 | 21.00 | 69.80 | 3.00 | 32.00 | 8.00 | 105.00 | 28.00 | 31.80 | 2.00 |
| 22 | BIC | 134.35 | 1.20 | 1.00 | 230.40 | 6.00 | 0.00 | 0.00 | 22.00 | 7.00 | 46.40 | 2.00 |
| 23 | BIC | 1260.55 | 37.20 | 12.00 | 37.00 | 6.00 | 2.80 | 1.00 | 74.00 | 22.00 | 149.00 | 5.00 |
| 24 | BIC | 72.71 | 0.00 | 0.00 | 289.80 | 5.00 | 0.00 | 0.00 | 10.20 | 4.00 | 0.00 | 0.00 |
| 25 | BIC | 1821.98 | 55.40 | 26.00 | 85.80 | 7.00 | 28.20 | 8.00 | 116.40 | 34.00 | 14.20 | 4.00 |
| 26 | BIC | 75.13 | 0.00 | 0.00 | 292.60 | 3.00 | 0.00 | 0.00 | 7.40 | 2.00 | 0.00 | 0.00 |
| 27 | BIC | 1011.25 | 31.80 | 17.00 | 112.80 | 4.00 | 15.00 | 4.00 | 65.80 | 24.00 | 74.60 | 4.00 |
| 28 | BIC | 454.03 | 7.60 | 4.00 | 5.00 | 2.00 | 0.00 | 0.00 | 26.00 | 9.00 | 261.40 | 7.00 |
| 29 | BIC | 525.09 | 6.60 | 3.00 | 247.40 | 3.00 | 9.60 | 1.00 | 14.00 | 4.00 | 22.40 | 3.00 |
| 30 | BIC | 347.55 | 12.80 | 5.00 | 250.60 | 15.00 | 0.00 | 0.00 | 36.60 | 19.00 | 0.00 | 0.00 |
| 31 | BIC | 74.35 | 0.00 | 0.00 | 300.00 | 1.00 | 0.00 | 0.00 | 0.00 | 0.00 | 0.00 | 0.00 |
| 32 | BIC | 1883.07 | 46.80 | 27.00 | 76.60 | 13.00 | 38.20 | 14.00 | 138.40 | 42.00 | 0.00 | 0.00 |

F. 26-30 min.

| Rat | Treatment | Traveled  distance (cm)  26-30 min | Ambulation  duration (s)  26-30 min | Ambulation  frequency (n)  26-30 min | Sitting  duration (s)  26-30 min | Sitting  frequency (n)  26-30 min | Rearing  duration (s)  26-30 min | Rearing  frequency (n)  26-30 min | Head-shoulder  motility duration (s)  26-30 min | Head-shoulder  motility frequency (n)  26-30 min | Grooming  duration (s)  26-30 min | Grooming  frequency (n)  26-30 min |
| --- | --- | --- | --- | --- | --- | --- | --- | --- | --- | --- | --- | --- |
| 1 | MUS | 72.86 | 0.00 | 0.00 | 297.80 | 2.00 | 0.00 | 0.00 | 1.60 | 1.00 | 0.00 | 0.00 |
| 2 | MUS | 1722.67 | 67.40 | 20.00 | 90.20 | 6.00 | 9.60 | 3.00 | 126.80 | 24.00 | 6.00 | 1.00 |
| 3 | MUS | 98.56 | 0.00 | 0.00 | 277.00 | 5.00 | 0.00 | 0.00 | 19.80 | 5.00 | 3.20 | 1.00 |
| 4 | MUS | 90.28 | 0.00 | 0.00 | 211.60 | 14.00 | 0.00 | 0.00 | 88.20 | 14.00 | 0.00 | 0.00 |
| 5 | MUS | 1077.15 | 61.80 | 20.00 | 34.20 | 4.00 | 5.20 | 2.00 | 198.80 | 24.00 | 0.00 | 0.00 |
| 6 | MUS | 219.76 | 2.20 | 1.00 | 174.40 | 3.00 | 0.00 | 0.00 | 12.80 | 3.00 | 110.60 | 1.00 |
| 7 | MUS | 57.10 | 0.00 | 0.00 | 190.80 | 2.00 | 0.00 | 0.00 | 0.00 | 0.00 | 109.20 | 1.00 |
| 8 | MUS | 151.10 | 0.00 | 0.00 | 257.80 | 3.00 | 0.00 | 0.00 | 2.20 | 1.00 | 40.00 | 1.00 |
| 9 | MUS | 970.82 | 39.00 | 13.00 | 78.20 | 9.00 | 18.00 | 2.00 | 164.80 | 23.00 | 0.00 | 0.00 |
| 10 | MUS | 157.00 | 6.00 | 3.00 | 258.60 | 8.00 | 0.00 | 0.00 | 35.40 | 10.00 | 0.00 | 0.00 |
| 11 | MUS | 1673.71 | 86.80 | 33.00 | 1.20 | 1.00 | 35.60 | 8.00 | 125.20 | 32.00 | 51.20 | 4.00 |
| 12 | MUS | 155.73 | 1.00 | 1.00 | 134.60 | 12.00 | 0.00 | 0.00 | 65.00 | 14.00 | 99.40 | 1.00 |
| 13. | MUS | 114.45 | 0.00 | 0.00 | 209.00 | 16.00 | 0.00 | 0.00 | 91.00 | 16.00 | 0.00 | 0.00 |
| 14 | MUS | 731.23 | 22.80 | 7.00 | 125.80 | 7.00 | 6.00 | 2.00 | 99.00 | 14.00 | 46.40 | 3.00 |
| 15 | MUS | 907.78 | 29.40 | 13.00 | 56.00 | 11.00 | 7.80 | 3.00 | 121.20 | 24.00 | 85.60 | 4.00 |
| 16 | MUS | 658.36 | 22.00 | 6.00 | 204.80 | 10.00 | 13.00 | 3.00 | 47.40 | 19.00 | 12.80 | 3.00 |
| 17 | BIC | 1111.91 | 31.00 | 13.00 | 33.00 | 7.00 | 21.20 | 10.00 | 112.80 | 25.00 | 102.00 | 5.00 |
| 18 | BIC | 309.10 | 1.80 | 1.00 | 183.80 | 18.00 | 0.00 | 0.00 | 64.20 | 26.00 | 50.20 | 9.00 |
| 19 | BIC | 1629.46 | 59.20 | 24.00 | 31.40 | 10.00 | 34.60 | 12.00 | 121.20 | 32.00 | 53.60 | 4.00 |
| 20 | BIC | 1153.23 | 40.80 | 14.00 | 67.00 | 23.00 | 1.60 | 1.00 | 51.80 | 25.00 | 138.80 | 16.00 |
| 21 | BIC | 949.65 | 27.20 | 16.00 | 43.40 | 7.00 | 12.60 | 7.00 | 71.20 | 22.00 | 145.60 | 3.00 |
| 22 | BIC | 332.56 | 16.20 | 5.00 | 189.80 | 8.00 | 0.00 | 0.00 | 29.60 | 9.00 | 64.40 | 7.00 |
| 23 | BIC | 754.49 | 15.40 | 7.00 | 24.40 | 2.00 | 7.80 | 2.00 | 37.60 | 12.00 | 214.80 | 7.00 |
| 24 | BIC | 101.09 | 0.00 | 0.00 | 291.80 | 5.00 | 0.00 | 0.00 | 8.20 | 4.00 | 0.00 | 0.00 |
| 25 | BIC | 735.43 | 21.00 | 9.00 | 68.00 | 3.00 | 0.00 | 0.00 | 48.60 | 15.00 | 162.40 | 5.00 |
| 26 | BIC | 38.23 | 0.00 | 0.00 | 300.00 | 1.00 | 0.00 | 0.00 | 0.00 | 0.00 | 0.00 | 0.00 |
| 27 | BIC | 398.73 | 25.00 | 4.00 | 130.20 | 6.00 | 0.00 | 0.00 | 12.20 | 5.00 | 132.60 | 2.00 |
| 28 | BIC | 1199.27 | 36.40 | 18.00 | 109.00 | 8.00 | 30.40 | 11.00 | 50.40 | 27.00 | 73.80 | 4.00 |
| 29 | BIC | 130.54 | 4.20 | 1.00 | 294.20 | 2.00 | 0.00 | 0.00 | 1.60 | 1.00 | 0.00 | 0.00 |
| 30 | BIC | 757.72 | 18.00 | 9.00 | 18.40 | 4.00 | 0.00 | 0.00 | 37.20 | 19.00 | 226.40 | 8.00 |
| 31 | BIC | 55.54 | 0.00 | 0.00 | 298.60 | 2.00 | 0.00 | 0.00 | 1.40 | 1.00 | 0.00 | 0.00 |
| 32 | BIC | 115.33 | 0.00 | 0.00 | 294.00 | 5.00 | 0.00 | 0.00 | 6.00 | 4.00 | 0.00 | 0.00 |
